# The ‘Oma’s of the Gammas—Cancerogenesis by γ-Herpesviruses

**DOI:** 10.3390/v16121928

**Published:** 2024-12-17

**Authors:** Anwesha Banerjee, Debashree Dass, Soumik Mukherjee, Mollina Kaul, R. Harshithkumar, Parikshit Bagchi, Anupam Mukherjee

**Affiliations:** 1 Division of Virology, ICMR-National Institute of Translational Virology and AIDS Research, Pune 411026, MH, India; banerjee.anwesha1991@gmail.com (A.B.); debashree650@gmail.com (D.D.); soumik.mukherjee2020@vitbhopal.ac.in (S.M.); mollinakaul@gmail.com (M.K.); harshith8398@gmail.com (R.H.); 2 Department of Molecular Microbiology, Washington University School of Medicine, St. Louis, MO 63110, USA; 3 AcSIR—Academy of Scientific & Innovative Research, Ghaziabad 201002, UP, India

**Keywords:** gamma herpesviruses, Epstein–Barr, Kaposi’s sarcoma, oncogene, epigenetic, antiviral, vaccine, lymphoma, co-infection, immune evasion

## Abstract

Epstein–Barr virus (EBV) and Kaposi’s sarcoma-associated herpesvirus (KSHV), which are the only members of the gamma(γ) herpesviruses, are oncogenic viruses that significantly contribute to the development of various human cancers, such as Burkitt’s lymphoma, nasopharyngeal carcinoma, Hodgkin’s lymphoma, Kaposi’s sarcoma, and primary effusion lymphoma. Oncogenesis triggered by γ-herpesviruses involves complex interactions between viral genetics, host cellular mechanisms, and immune evasion strategies. At the genetic level, crucial viral oncogenes participate in the disruption of cell signaling, leading to uncontrolled proliferation and inhibition of apoptosis. These viral proteins can modulate several cellular pathways, including the NF-κB and JAK/STAT pathways, which play essential roles in cell survival and inflammation. Epigenetic modifications further contribute to EBV- and KSHV-mediated cancerogenesis. Both EBV and KSHV manipulate host cell DNA methylation, histone modification, and chromatin remodeling, the interplay of which contribute to the elevation of oncogene expression and the silencing of the tumor suppressor genes. Immune factors also play a pivotal role in the development of cancer. The γ-herpesviruses have evolved intricate immune evasion strategies, including the manipulation of the major histocompatibility complex (MHC) and the release of cytokines, allowing infected cells to evade immune detection and destruction. In addition, a compromised immune system, such as in HIV/AIDS patients, significantly increases the risk of cancers associated with EBV and KSHV. This review aims to provide a comprehensive overview of the genetic, epigenetic, and immune mechanisms by which γ-herpesviruses drive cancerogenesis, highlighting key molecular pathways and potential therapeutic targets.

## 1. Introduction

Similar to other herpesviruses, gamma (γ)-herpesviruses are double-stranded DNA viruses capable of inducing persistent infections. However, γ-herpesviruses are distinct from alpha- and beta-herpesviruses in their efficiency in triggering numerous malignancies, especially in immunocompromised patients. Being lymphotropic in nature, establishing latency mainly in the B cells, these viruses are associated with the development of lymphoproliferative diseases and lymphomas, as well as other nonlymphoid cancers [1].

Also known as the human herpesvirus 4 (HHV4), the Epstein–Barr virus or EBV is omnipresent in nature, causing infections in about 90% of the global adult population. Although the virus is commonly transmitted orally amongst children and adolescents, the age at which these primary infections are acquired has been rising in developed countries owing to their greater socioeconomic status [2,3]. EBV has a genome size of ~172Kb. It is an oncovirus, with its associated cancers following distinct geographic distributions owing to the variations in the phylogeographical attributes of the various EBV strains [4,5]. The differences in the genetic sequences allowed the EBV to be classified into subcategories 1 and 2 or EBV type A and B. The global prevalence of type 1 EBV is more than that of EBV type 2, with type 2 being more common in Africa, Alaska, and Papua New Guinea rather than in Asia, Europe, and the USA [6,7]. Lately, the EBV strains have been segregated into 12 groups, with each of these groups portraying distinct EBV-related disease and geographic patterns [8]. Amongst other factors, ethnicity, nationality, lifestyle, and the presence of single-nucleotide polymorphisms are some of the most crucial ones contributing to the specific geographical patterns of the cancers associated with EBV. Having said that, the risk factors governing cancerogenesis in a fraction of the population, while considerately exempting a major part of the global population, are diverse and predominantly affected by genetics and the environment [9].

Another representative of the gamma herpesvirus family, the Kaposi’s sarcoma-associated herpesvirus (KSHV) or HHV-8, which has a genome size of ~165 Kb and encodes about 100 genes, was first identified 27 years ago in a lesion from a patient with AIDS-associated Kaposi’s sarcoma (KS). Therefore, the relationship between KSHV infection and the development of KSHV-associated tumors, including KS and primary effusion lymphoma (PEL), has become one of the most well-established among human tumor viruses since the discovery of the virus. Primarily transmitted sexually, HHV-8 is prevalent in MSM (men who have sex with men) population [10]. KSHV is consistently found in all KS and PEL samples. KS is a malignancy of endothelial cells that is characterized by highly vascularized lesions affecting the skin and mucosal tissues. KSHV-induced diseases primarily occur in immunocompromised individuals. AIDS-associated KS, also known as epidemic KS and iatrogenic KS, which arise due to immunosuppressive therapies following organ transplantation, are the most common forms linked to immunosuppression. However, subtypes of KS can occur in individuals without evident immunosuppression, such as classical KS, which predominantly affects older men of Mediterranean descent, and endemic KS, common in African adults and children. In fact, the strong association of KS with immunosuppression led to its prominence during the AIDS epidemic, ultimately resulting in the discovery of KSHV. While the introduction of antiretroviral therapy (ART) has significantly reduced the incidence of KS in the United States, the number of new cases has plateaued over the past decade, with approximately 900 cases reported annually [11,12]. This review gives an elaborate account of the cancers associated with the γ-herpesviruses and the factors that play a vital role in cancerogenesis.

## 2. Molecular Virology of EBV and KSHV Infection Leading to Cancerogenesis

The EBV viral proteins, such as the nuclear antigens (EBNA1, EBNA2, and EBNA3), the latent membrane proteins (LMP1 and LMP2), and the KSHV proteins vFLIP, vCyclin, kaposins, and LANA, along with viral miRNAs, are essential for transforming primary B cells into lymphoblastoid cells, leading to malignancy [13,14]. These proteins and miRNAs aid in viral replication, evading host immune responses, preventing apoptosis in infected cells, and supporting cell survival, thereby promoting cancer development upon infection. Also, epigenetic regulators participating in the immortalization of cells after infection, even in the latent state, have also been brought to light [15,16]. Therefore, it can be fervently deduced that these γ-herpesviruses come fully equipped to establish tumorigenesis in the host.

### 2.1. Genetic Factors Leading to Cancer

#### 2.1.1. Role of Viral Oncogenes

**Figure 1:**
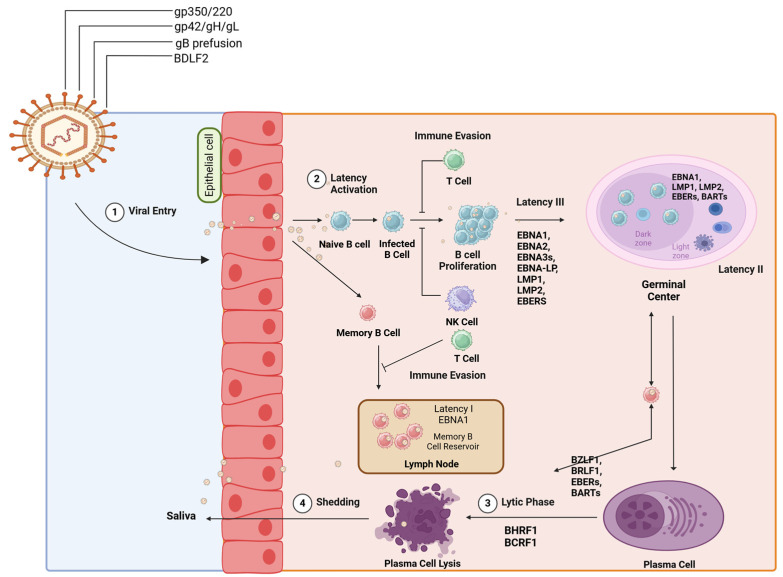
Life cycle of Epstein–Barr virus (EBV). This figure depicts the infection and persistence mechanism of Epstein–Barr virus (EBV). The process is illustrated in several stages. (1) Viral entry—EBV enters the epithelial cells using viral glycoproteins (gp350/220, gp42/gH/gL, gB prefusion, BDLF2). (2) Latency activation—the virus infects B cells, leading to their proliferation and the establishment of latent infection. (3) Lytic phase—in some cells, EBV enters the lytic phase, resulting in the production of new viral particles and plasma cell lysis. (4) Shedding—viral particles are shed into saliva, facilitating transmission. The figure also illustrates different latency stages (I, II, III) and the associated viral proteins (EBNA1, EBNA2, EBNA3s, LMP1, LMP2). It shows how EBV evades the immune system by manipulating T cells and NK cells. The germinal center reaction and the establishment of a memory B cell reservoir in lymph nodes are also depicted, highlighting the lifecycle of EBV and its ability to persist in the host.

The oncogenic potential of EBV is driven by a combination of viral gene expression, host genetic susceptibility, and epigenetic alterations, disrupting normal cellular processes and leading to malignancies, such as gastric carcinomas (GC), nasopharyngeal carcinoma (NPC), Burkitt’s lymphoma (BL), and post-transplant lymphomas, arising [17]. EBV employs several oncogenic mechanisms to promote cell transformation and cancer development. EBV encodes viral proteins, EBNA1, EBNA2 and EBNA3, LMP1, and LMP2, which are essential for transforming primary B cells into lymphoblastoid cells, leading to malignancy [13]. An illustrative account of the involvement of EBV viral proteins in the EBV life cycle can be found in Figure 1.

EBNA1 binds to the plasmid origin of replication (oriP) through its C terminus to ensure EBV genome replication, and the N terminus of the EBNA1 allows EBV to attach to an arbitrary location on the infected cells for proper distribution of viral genome during cell division [18,19]. EBNA1 also modulates telomere regulation in EBV-infected cells, and it also activates transcription of viral and host genes, which positively regulates the expressions of active contributors in cancer, such as LMPs and the enzyme NOX2, while evading immune detection [20,21]. Therefore, EBNA1 maintains the viral genome in infected cells and can inhibit apoptosis, supporting cell survival and leading to oncogenesis. EBNA2 functions as a transcription factor, regulating viral and cellular gene expression, including LMPs and MYC, which are crucial for B-cell transformation and survival [22]. EBNA-LP, in conjunction with EBNA2, which are the first latent proteins to be discovered following EBV infection, activates viral and cellular genes and is crucial for the transformation of B cells. The EBNA3 protein family includes EBNA3A, 3B, and 3C. For B-cell transformation in vitro, EBNA3A and EBNA3C are required, while EBNA3B is not necessary [23]. EBNA3A and EBNA3C are involved in suppressing p16INK4a and the pro-apoptotic protein BIM (BCL2L11) [24,25]. These proteins maintain the EBV-infected lymphoblastoid cells in a proliferative state, preventing their differentiation into plasma cells. The function of EBNA3 protein involves interacting with cellular epigenetic regulators and corepressors, such as Polycomb repressor proteins and, potentially, histone deacetylase complexes, including the SIN3A complex [26]. LMP1 mimics CD40 signaling [27]. It has multiple transmembrane-spanning domains and a carboxyl terminus, which can interact with a number of tumor necrosis factor receptor-associated factors (TRAFs) [28]. In LMP1-expressing epithelial and B cells upon EBV infection, this interaction causes activation of NF-κB, JNK, and MAPK pathways [29]. The activation of these pathways leads to the induction of anti-apoptotic proteins, increased expression of adhesion molecules, and enhanced cell survival. These effects collectively contribute to the transformation of EBV-infected cells and their resistance to apoptosis [30]. LMP1 induces Bcl-2 family members, c-FLIP, c-IAPs, and adhesion molecules through NF-κB to deliver survival signals [31,32]. In addition, LMP1 stimulates the production of matrix metalloproteinase-9 (MMP-9) and fibroblast growth factor-2 (FGF-2) [33,34]. Another important EBV protein, LMP2, provides additional survival signals to infected cells by manipulating B-cell receptor signaling [35]. This manipulation helps maintain viral latency and promotes the survival of infected B cells, even in the absence of normal B-cell stimulation. LMP-2A, by interacting with Lyn and Syk, imitates B-cell receptor (BCR) signaling, which involves activating the survival pathway PI3K/AKT, which restricts apoptosis and promotes cell survival [36]. LMP-2A is strongly linked to lymphoma development, as it encodes for the activation of B-cell receptor (BCR) signaling independent of antigen molecules. This activation subsequently triggers the transactivation of the HERV-K18, leading to a significant T-cell response [37]. Upon EBV infection, other viral proteins, such as BRLF1 and BZLF1, are produced during the lytic phase, leading to the expression of viral proteins like BILF1, BMRF1, and BNLF2A, which play important roles in cell survival and maintaining malignancies [38]. EBV also encodes a viral homolog of the cellular anti-apoptotic protein Bcl-2, called BHRF1 [39]. This protein helps infected cells evade apoptosis, further contributing to cell survival and potential transformation. The latent phase of EBV also leads to the expression of non-coding RNAs (ncRNAs), which play a role in maintaining the viral infection and are associated with EBV-related cancers [40].

**Figure 2:**
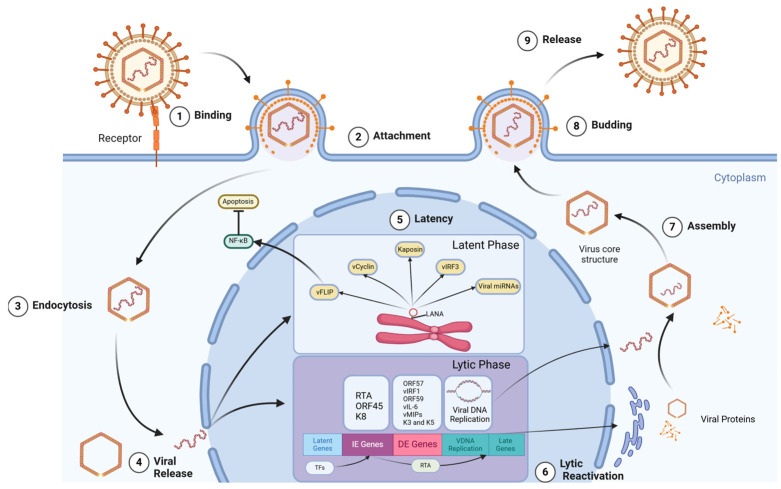
Life cycle of Kaposi’s sarcoma-associated herpesvirus (KSHV)**.** This figure illustrates the life cycle of Kaposi’s sarcoma-associated herpesvirus (KSHV). The process is divided into several key stages: (1) Binding—KSHV attaches to a cell receptor. (2) Attachment—the virus adheres to the cell membrane. (3) Endocytosis—the virus enters the cell through endocytosis. (4) Viral release—the viral capsid is released into the cytoplasm. (5) Latency—KSHV enters a latent phase, where LANA plays a crucial role in maintaining the viral genome and regulating host cell processes. (6) Lytic reactivation—the virus switches to the lytic phase, where viral DNA replication and gene expression occur. (7) Assembly—new virus particles are assembled. (8) Budding—mature virions bud from the cell membrane. (9) Release—new viral particles are released to infect other cells. The image also shows key viral proteins and their functions in both latent (vFLIP, vCyclin, kaposin, vIRF3) and lytic (RTA, ORF45, K8) phases. The figure demonstrates how KSHV manipulates cellular processes like apoptosis and NF-κB signaling to ensure its survival and replication.

KSHV employs several oncogenic mechanisms that parallel those of EBV but with its own unique set of viral proteins. Key latency-expressed proteins that may contribute to cellular transformation include the *kaposin/K12* gene, viral cyclin *(v-cyclin, ORF72)*, viral FLICE inhibitory protein *(vFLIP, ORF71)*, and *LANA (ORF73)* [41]. One of the key players in KSHV-mediated oncogenesis is the latency-associated nuclear antigen (LANA). LANA is essential for maintaining the viral episome during latent infection and also interferes with important cell cycle regulators, such as p53 and pRb [42]. LANA promotes cell growth and survival by suppressing the activity of p53 activity because p53 causes cell death upon KSHV infection [43]. The viral FLICE inhibitory protein (vFLIP) is another important KSHV oncoprotein and a potent activator of the NF-κB pathway, which promotes cell survival and proliferation [44]. Additionally, vFLIP inhibits apoptosis by interfering with the death receptor signaling pathway, further enhancing the survival of KSHV-infected cells [45]. KSHV also encodes a viral cyclin (v-cyclin) that deregulates the cell cycle. V-cyclin forms complexes with cyclin-dependent kinase 6 (CDK6), leading to the phosphorylation of pRb and other targets [46]. This activity promotes progression through the G1/S phase of the cell cycle, contributing to uncontrolled cell proliferation. Moreover, vFLIP mediates pro-survival signaling, while kaposins promote cytokine production and cell growth [47]. Additionally, vFLIP modulates the production of human interleukin-6 (hIL-6), which is upregulated with the assistance of kaposin B and vGPCR, leading to B lymphocyte proliferation [48]. Viral IL-6 (vIL-6) produced by KSHV also upregulates VEGF expression, activating angiogenesis [14]. Increased cytokine levels, particularly IL-6, are associated with poor prognosis in PEL patients, with many meeting the criteria for KICS [49]. MCD, a polyclonal tumor, heavily depends on cytokines like human IL-6 [50]. Mouse cell lines consistently expressing vIL-6 generate large amounts of VEGF and are carcinogenic in nude mice [51]. vGPCR can stimulate angiogenesis and cell proliferation through various signaling pathways, contributing to Kaposi’s sarcoma development [52]. In addition to latent proteins, many KS tumors and PEL grafts produce lytic viral genes, possibly resulting from incomplete or abortive viral reactivation [53]. These genes include the KSHV interferon regulatory factor (vIRF-1), G-coupled receptor (vGPCR) homologs, and constitutive signaling proteins like K1 and K15 [54]. The K1 ORF encodes the variable ITAM-containing protein (VIP), which has the potential to transform rat fibroblasts. These transformed cells can cause multiple, widespread tumors when injected into nude mice [55]. Additionally, transgenic animals expressing VIP develop sarcomas and lymphomas, and VIP can functionally replace the saimiri transformation protein (STP) of herpesvirus saimiri (HVS) to induce lymphomas in common marmoset monkeys [56]. VIP can directly alter cells and induce inflammatory cytokines and angiogenic factors [57]. The K15 ORF-encoded latency-associated membrane protein (LAMP) is involved in mitogenic and survival signaling through Src-family kinases and NF-κB activation [58]. It may also enhance cell survival by associating with the Bcl-2-related anti-apoptotic protein HAX-1 [59]. K1 and K15 contribute to cellular transformation through various signaling pathways. K1 activates the PI3K/AKT pathway, promoting cell survival and angiogenesis, while K15 activates the NF-κB and MAPK pathways, contributing to cell proliferation and survival [60]. The viral interferon regulatory factors (vIRFs) interfere with normal interferon signaling, helping the virus evade the host’s innate immune response. For example, vIRF-3 is specific to B cells, while vIRF-1 is specific to endothelial cells [61]. The interplay between latent and lytic viral proteins, along with the modulation of host cytokine production (especially IL-6), contributes significantly to KSHV-associated malignancies. The viral proteins involved in the life cycle of KSHV are displayed in Figure 2. Therefore, both latent and certain lytic genes can be considered targets for tumor-specific therapy in KS.

#### 2.1.2. Role of Viral microRNAs (miRNAs) in Cancer

EBV encodes two clusters of 44 mature miRNAs: the BamHI A rightward transcript (BART) cluster and the BamHI H rightward open reading frame 1 (BHRF1) cluster [62]. The BART cluster, which includes genes such as BARF0, RPMS1, RPMS1A, and A73, is particularly important in EBV biology [63]. These genes do not produce any proteins but are known to modulate cell growth by regulating the expression of host genes. The miR-BART expression profiles, when investigated in clinical samples from EBV-positive gastric cancer patients, showed miR-BART7-3p with the highest expression, followed by miR-BART9-3p, miR-BART1-3p, and miR-BART5-5p [63]. miR-BART17-5p has been found in both gastric cancer tissue and EBV-positive gastric cancer cell lines. High serum levels of miR-BART17-5p were associated with disease progression and recurrence in patients with nasopharyngeal carcinoma. Additionally, miR-BHRF-1-1 is detected in all samples of EBV-associated primary central nervous system post-transplant lymphoproliferative disorder (PTLD), whereas miR-BHRF-1-2 is present in around half of the patients [64]. BART miRNAs target a wide range of host genes involved in apoptosis regulation and immune modulation [65]. miR-BART5 targets the pro-apoptotic protein PUMA, promoting cell survival by inhibiting apoptosis [66]. Several miR-BARTs have been shown to reduce the production of BIM, a pro-apoptotic protein. Other miRNA-BARTs, such as miR-BART3-3p, miR-BART5-5p, and miR-BART2-5p, appear to help gastric tumor cells evade the immune system. Additionally, miR-BHRF1-3 and miR-BART17 target TAP2, which prevents antigen processing and presentation on MHC-class I+ cells. miR-BHRF1-2-5p inhibits the synthesis of IL-1R1, a protein responsible for alerting the immune system to viral infections [67]. Several miRNAs, including BHRF1-2, BART1-5p, BART1-3p, and BART2-5p, target antigen-processing genes [68]. Furthermore, miR-BART6-3p targets RIG-I, which inhibits innate immune responses. Similarly, miR-BART20-5p and miR-BART8 target IFN-γ and STAT1, respectively, reducing cellular immunity against tumor cells. miR-BART16 blocks IFN signaling by targeting CBP. miR-BART15-3p suppresses the manufacture of IL-1β and IL-18 by targeting NLRP3. miR-BART5-5p targets TP53, which promotes cell survival. miR-BART15-5p plays a role in limiting inflammation, which may facilitate EBV infection [69]. It may also induce apoptosis by targeting the inhibitory proteins of apoptosis, such as BRUCE and TAX1BP1. This could lead to the death of tumor cells and the surrounding cells, including immune cells, through exosome release. According to Li C.W. and his group, the EBV virus miR-BART14 was discovered to suppress the expression of lncRNA AFG3L1P. By targeting AFG3L1P, miR-BART14 may contribute to cell survival and mitochondrial dysfunction, both of which are hallmarks of cancer. The miR-BART cluster has also been shown to regulate epithelial–mesenchymal transition (EMT) and metastasis. miR-BART6-3p targets the long non-coding RNA LOC553103, inhibiting epithelial–mesenchymal transition and metastasis in nasopharyngeal carcinoma [70]. In nasopharyngeal cancer (NPC), miR-BART-22 has been found to be significantly upregulated and correlates with tumor progression and poor prognosis. It promotes EMT and metastasis by targeting MOSPD2 mRNA and activating the Wnt/β-catenin signaling pathway [71]. According to studies, miR-BART7-3p may promote tumor growth and invasion by inhibiting proteins such as PTEN and SMAD7 [72]. Additionally, SMAD7 inhibition may produce cancer stem cell-like features, potentially increasing resistance to chemoradiotherapy. In an animal model, gold nanoparticles with an anti-miR-BART7-3p antibody could inhibit tumor growth, emphasizing their potential role in epithelial malignancies [73]. Therefore, BART miRNAs are involved in reprogramming the transcription of gene expression of host cells in Epstein–Barr virus-associated carcinomas (Table 1).

On the other hand, KSHV encodes a cluster of 12 pre-miRNAs that give rise to 25 mature miRNAs [74]. These miRNAs play crucial roles in cell cycle control, angiogenesis, and immune evasion. The mature KSHV miRNAs are named miR-K12-1 to miR-K12-12, or simply miR-K1 to miR-K12, based on their proximity to the kaposin (K12) gene [75]. However, studies have shown that the individual KSHV miRNAs are expressed at dramatically different copy numbers. While some miRNAs like miR-K1, miR-K3, miR-K4-3p, miR-K6-3p, and miR-K11 are consistently and abundantly expressed, others such as miR-K9 are likely to be present at much lower levels. This differential expression likely results from differences in processing efficiency, RISC loading, and/or stability, despite being derived from common pri-miRNA transcripts [76]. The expression levels of KSHV miRNAs also vary across different primary effusion lymphoma (PEL) cell lines and in de novo infection models. Interestingly, some KSHV miRNAs have evolved to mimic host miRNAs. KSHV miR-K12-11 is an ortholog of cellular miR-155, which is known to play roles in B-cell development and lymphomagenesis. This mimicry allows the virus to exploit existing cellular regulatory networks to its advantage [77]. MIR17HG, MIR155HG, MALAT1, and AFAP1-AS1 are targets of KSHV miR-K12-11 [78]. Other KSHV miRNAs like miR-K12-10 target TWEAKR, which is involved in apoptosis, promoting cell survival and potentially contributing to the resistance of KSHV-infected cells to apoptotic stimuli [79]. KSHV-associated malignancies, particularly Kaposi’s sarcoma, are characterized by abnormal angiogenesis. miR-K1, K3-3p, K6-3p, and K11 promote angiogenesis by targeting anti-angiogenic factors like thrombospondin 1 [80]. Other miRNAs like miR-K6-5p enhance endothelial cell tubulogenesis [81]. These miRNAs also promote cell migration and invasion, contributing to tumor dissemination. KSHV miRNAs manipulate the host cell cycle and apoptotic pathways to create an environment promoting viral replication and oncogenesis. miR-K1 regulates the cell cycle by targeting p21, a key cell cycle inhibitor [82]. Several miRNAs, including miR-K1, K3, and K4-3p inhibit apoptosis by targeting caspase 3 [83]. This regulation of cell survival and proliferation is crucial for maintaining the population of infected cells. Thus, the oncogenic potential of KSHV is partly attributed to its miRNAs (Table 1). A cluster of KSHV miRNAs is essential for cellular transformation. KSHV miRNAs achieve this by targeting multiple cancer-related pathways. miR-K10a and K10b promote cell survival by modulating TGF-β signaling [84]. miR-K11 and miR-K6 influence the differentiation state of endothelial cells, contributing to the unique vascular tumors associated with KSHV [85]. KSHV miRNAs also play a role in regulating host cell metabolism. KSHV miRNAs can induce the Warburg effect, a hallmark of cancer metabolism. However, they can also suppress aerobic glycolysis under certain conditions, demonstrating their ability to fine-tune cellular metabolism to suit viral needs and the tumor microenvironment [86]. Additionally, γ-herpesviruses are capable of employing epigenetic modification strategies to support their self-pathogenesis and the survival of their host cells, thereby ensuring their own.

### 2.2. Epigenetic Involvement Leading to Cancer

The core epigenetic strategies that drive host–virus interactions and are essential for viral replication and the progression of EBV- and KSHV-associated cancer include the association with histone modifications, modification of DNA methylation patterns in both the host and viral genomes, and the microRNA targeting of the host cell factors [87].

#### 2.2.1. Methylation, Histone Modifications, and Chromatin Remodeling of Host Genome

EBV infection leads to epigenetic changes in host cells, which is the primary cause of a number of autoimmune diseases and malignancies [88,89,90]. A hallmark of EBV-associated cancers is the hypermethylation of tumor suppressor gene promoters, such as p16INK4A, E-cadherin, and PTEN [91]. EBV latent proteins, such as EBNA1 and LMP1, interact with host DNA methyltransferases (DNMTs), leading to the hypermethylation of these genes and resulting in their transcriptional silencing, promoting uncontrolled cell growth, and contributing to the cancer phenotype [92]. Simultaneously, EBV infection can cause global hypomethylation, leading to increased genomic instability, activation of oncogenes, and altered chromosomal organization [93]. Additionally, LMP2A upregulates the production of DNMT, which leads to abnormal methylation patterns [94]. The interplay between localized hypermethylation and global hypomethylation leads to an epigenetic environment that induces oncogenesis. Histone acetylation is another key modification affected by EBV. The viral protein EBNA3C interacts with histone deacetylases (HDACs), promoting deacetylation and the silencing of key cellular genes [23]. A prime example is the repression of the p16INK4A locus through the recruitment of HDAC1 and HDAC2 by EBNA3C [95]. Other EBV proteins, such as LMP1 and EBNA2, are also directly involved in histone modifications [96,97]. LMP1 induces expression of DNMTs and enhances global DNA methylation [98]. LMP1 also influences histone methylation by upregulating histone methyltransferases like EZH2, resulting in the trimethylation of H3K27 and the repression of genes involved in apoptosis and immune recognition [96]. EBNA2 recruits histone acetyltransferases (HATs) to activate both viral and cellular promoters. This also leads to increased acetylation and transcriptional activation of the genes involved in cell proliferation, such as *c-MYC* and *cyclin D2* [99]. These histone modifications work in concert to create a chromatin environment that favors viral persistence and cellular transformation. EBV infection leads to significant changes in chromatin structure, affecting both the viral and host genomes. The virus alters nucleosome positioning around key regulatory elements, as seen in the repositioning of nucleosomes at the Cp promoter during the latency phase. This repositioning is important for the regulation of EBNA expression. EBV infection also promotes the formation of repressive chromatin domains around silenced tumor suppressor genes [100]. Together with the other epigenetic regulators covered here, miRNAs also aid in host–virus interaction, leading to tumorigenic, autoimmune, and infection-related illnesses [88,89,90]. The variety of host targets in miRNA-based epigenetic regulation is highlighted by BART miRNAs [63]. Members of the BART miRNA family target RIG1, an inducible gene expressed in response to retinoic acid, in order to decrease innate immune response signaling. BART miRNAs further reduce immunity against tumors by binding to inflammasome proteins to prevent the production of IL-1β and IL-18, targeting transcriptional coactivators to prevent type I IFN signaling, and targeting STAT1 and IFN-γ to modify transduction in their respective signaling pathways [63].

The KSHV latency-associated nuclear antigen (LANA) promoter region is typically hypomethylated during latency, allowing for continuous expression of this essential latency protein [101]. LANA disrupts cell cycle checkpoints and promotes malignant transformation by inducing the hypermethylation and suppression of tumor suppressor genes including p53 and RB [102]. The hypermethylation of tumor suppressor gene promoters is one of the most important effects. For example, KSHV infection has been associated with increased methylation of the p16INK4A promoter in Kaposi’s sarcoma lesions [103]. This hypermethylation leads to a silencing of p16INK4A, a cell cycle regulator, thereby promoting uncontrolled cell proliferation. This epigenetic silencing of immune response genes helps the virus evade host immune detection and promotes viral replication. Conversely, KSHV infection can lead to the hypomethylation and activation of certain cellular genes that promote survival and proliferation. The promoter of the human telomerase reverse transcriptase (hTERT) gene is often hypomethylated in KSHV-infected cells, leading to increased telomerase activity and cellular immortalization [104]. Additionally, the activation of angiogenic factors, by which the vascular-rich phenotype Kaposi’s sarcoma lesions are characterized, is facilitated by hypomethylation and the consequent activation of genes encoding angiogenic agents, such as VEGF [105]. KSHV-LANA also plays a crucial role in modifying the host epigenetic landscape. LANA has been shown to interact with various histone-modifying enzymes, including histone deacetylases (HDACs) and the polycomb repressive complex 2 (PRC2) [106]. Through these interactions, LANA can induce repressive histone modifications at specific host gene promoters, leading to their silencing. LANA-mediated recruitment of PRC2 results in increased H3K27me3 at the promoters of various tumor suppressor genes, contributing to their epigenetic silencing (Toth et al., 2016). This mechanism has been observed for genes like p16INK4A and E-cadherin, promoting cell cycle progression and the loss of cell–cell adhesion, respectively. Conversely, KSHV infection can also lead to the activation of certain host genes through histone modifications. The viral protein K-Rta has been shown to interact with histone acetyltransferases, promoting histone acetylation and activation of both the viral and cellular promoters involved in lytic replication and cellular transformation [107]. KSHV also interacts with and modulates the activity of host chromatin-remodeling complexes. It has been shown to hijack the SWI/SNF chromatin-remodeling complexes to regulate both viral and cellular gene expression [108]. This interaction promotes the specific chromatin states that maintain viral replication and cellular transformation. Additionally, KSHV infection alters the distribution of chromatin boundary elements, such as CTCF binding sites, across the host genome [109]. This redistribution can lead to the disruption of normal gene regulation patterns and contribute to the aberrant gene expression observed in KSHV-associated malignancies.

#### 2.2.2. Interplay Between the Epigenetic Modifications

EBV highlights an interaction between different epigenetic mechanisms to maintain its oncogenic effects. Methylated DNA recruits methyl-CpG-binding proteins, which in turn recruit histone-modifying enzymes [110]. For example, methyl-CpG-binding protein 2 (MeCP2) is recruited to methylated promoters, leading to histone deacetylation and gene silencing [111]. ATP-dependent chromatin-remodeling complexes work in concert with histone-modifying enzymes to alter the chromatin structure and gene expression [112]. Furthermore, the recruitment of the SWI/SNF complex by EBNA2 leads to histone acetylation and the transcriptional activation of both viral and cellular genes [113]. EBV undergoes epigenetic modifications to regulate its latency and lytic cycles. Latency is triggered when the methylation of the viral genome silences lytic genes, maintaining latency [114]. Reactivation stimuli can lead to demethylation and chromatin remodeling, triggering the lytic cycle and contributing to oncogenesis through the expression of lytic proteins [115]. Similarly, KSHV coordinates DNA methylation, histone modifications, and chromatin remodeling to create an epigenetic landscape that favors an oncogenic environment [116]. Similar to EBV, the methylation of specific CpG islands in the host genome upon KSHV infection can also recruit methyl-CpG-binding proteins, which in turn attract histone-modifying enzymes and chromatin-remodeling complexes [117]. This cascade of events can lead to the formation of repressive chromatin domains around key tumor suppressor genes, silencing their expression. Similarly, KSHV proteins like LANA can simultaneously interact with DNA methyltransferases, histone-modifying enzymes, and chromatin-remodeling complexes [118]. These interactions allow for coordinated epigenetic regulation of both viral and cellular genes, leading to a cellular environment that is conducive to viral persistence and oncogenesis.

## 3. Immunological Alterations Supporting Oncogenesis by EBV and KSHV

**Figure 3:**
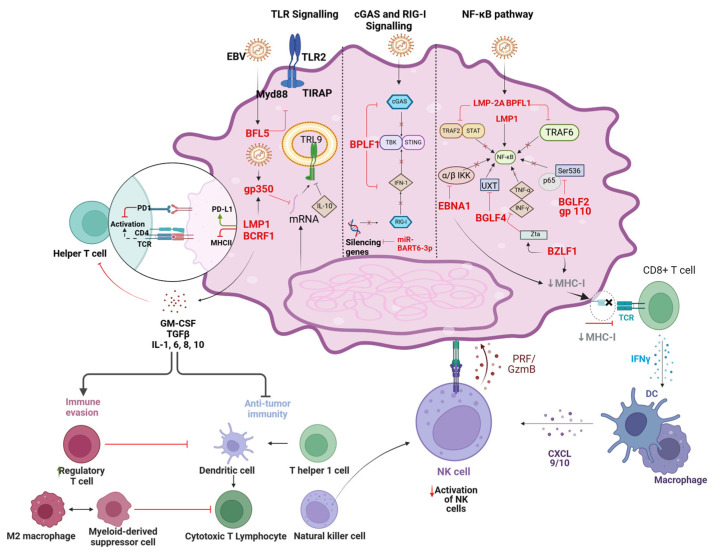
Immune evasion mechanisms of EBV. This figure illustrates the immune evasion strategies employed by Epstein–Barr virus (EBV) showing various cellular pathways and their manipulation by the virus. The figure depicts three main signaling pathways: TLR signaling, cGAS and RIG-I signaling, and the NF-κB pathway. EBV proteins (in red) such as LMP1, LMP2A, EBNA1, and BGLF4 interfere with these pathways to promote viral survival. The figure also shows how EBV modulates cell surface markers like PD-L1 and MHC-I to evade T-cell recognition. Key viral strategies include silencing genes, manipulating cytokine production, and interfering with antiviral responses. The bottom of the figure illustrates the broader immune landscape, showing how EBV influences various immune cells, including helper T cells, cytotoxic T cells, NK cells, dendritic cells, and macrophages. It highlights the balance between immune evasion and anti-tumor immunity, with EBV tipping the scales towards evasion through mechanisms like increasing regulatory T cells and myeloid-derived suppressor cells while inhibiting cytotoxic T lymphocytes and NK cell activation. Overall, the figure provides insights into how EBV subverts multiple aspects of the host immune response to establish persistent infection and potentially contribute to oncogenesis.

**Figure 4:**
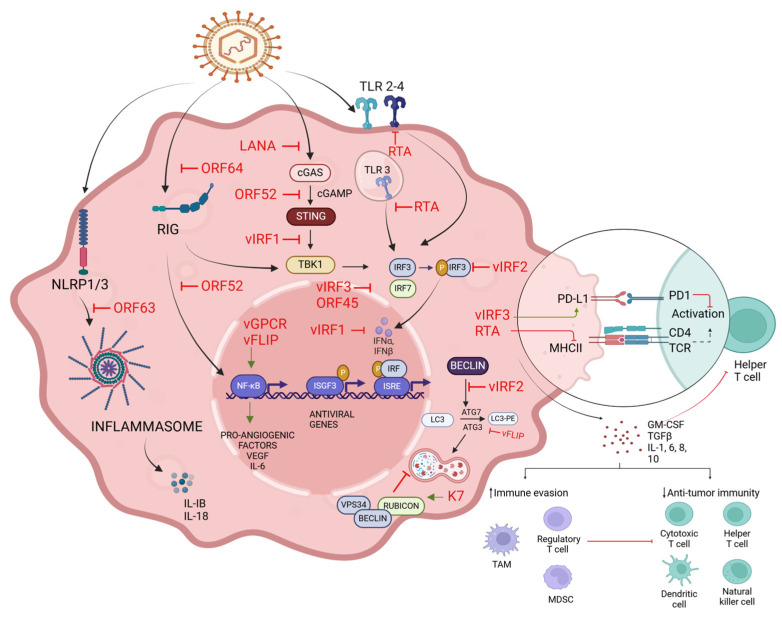
Immune evasion mechanisms of KSHV. This figure illustrates the immune evasion strategies employed by Kaposi’s sarcoma-associated herpesvirus (KSHV). It shows a KSHV-infected cell (red) interacting with immune cells, particularly a helper T cell. The figure depicts viral proteins (in red) interfering with host cell receptors, signaling pathways, and transcription factors. Key cellular components like the inflammasome, autophagy machinery, and antiviral gene expression are shown. The figure also highlights how KSHV modulates cell surface markers like PD-L1 and MHC II to evade T-cell recognition. Various immune cells in the tumor microenvironment are represented at the bottom, including regulatory T cells, cytotoxic T cells, and natural killer cells. Cytokines and growth factors involved in immune modulation and angiogenesis are also indicated. Overall, the figure demonstrates that KSHV is used to subvert the host immune response and promote its survival and potential oncogenesis.

The Epstein–Barr virus (EBV) is widely recognized as one of the most successful human pathogens due to its ability to establish latency in host cells and evade immune surveillance through sophisticated mechanisms [119,120,121,122]. The potent ability to evade the host immune system is crucial for EBV to establish persistent infections and, consequently, in the development of cancer [26,123,124] (Figure 3). Similarly, KSHV has evolved immune evasion strategies that contribute to tumorigenesis by allowing the virus to persist within host cells and escape immune surveillance. [125,126] (Figure 4). Through the manipulation of autophagy, interference with antigen presentation, modulation of cytokine production, and evasion of cytoplasmic DNA sensing, these viruses create an environment conducive to persistent infection and tumor development.

EBV has evolved mechanisms to exploit host cell autophagy for its replication and immune evasion. During its lytic cycle, EBV initially induces autophagy but subsequently inhibits this process through early lytic proteins. The recruitment of autophagic membranes by viral capsid proteins (e.g., BVRF2, BdRF1) facilitates envelope acquisition for the virion [127]. EBV manipulation of autophagy is further demonstrated by the accumulation of LC3-coated membranes during the lytic replication in B cells and the impairment of viral replication when early autophagy is inhibited. The virus appears to block autophagic flux during the late stages, as indicated by stable LC3 lipidation levels in the presence of bafilomycin and reduced colocalization of autophagosomes with lysosomes [128]. This strategic manipulation allows EBV to use autophagic machinery for intracellular transport while avoiding degradation. Inhibition of autophagic membrane formation leads to a cytosolic accumulation of viral DNA, suggesting its importance in viral envelope acquisition [129]. Conversely, stimulating autophagy enhances virus production. These findings not only highlight the interplay between EBV and cellular autophagy but also suggest potential therapeutic approaches, such as using autophagy inhibitors to enhance oncolytic viral therapy for EBV-related lymphomas.

KSHV also employs multiple mechanisms to manipulate host cell autophagy for its benefit and immune evasion. It expresses several proteins that interfere with different stages of the autophagy process. vFLIP prevents autophagy protein 3 (ATG3) from processing LC3 during phagosome elongation, thereby inhibiting autophagosome formation [130]. Another viral protein, K7, interacts with Rubicon to inhibit the fusion of autophagosomes with lysosomes, further disrupting the autophagic flux [131]. KSHV manipulates autophagy, which is linked to its oncogenic potential. Latent KSHV infection triggers the DNA damage responses (DDRs) that are characteristic of oncogene-induced senescence (OIS), yet infected cells show only modest autophagy levels and fail to senesce [132]. This aberrant response is maintained by the combined activities of the viral proteins v-cyclin and v-FLIP. While v-cyclin deregulates the cell cycle and triggers DDRs that could potentially promote autophagy, v-FLIP counteracts these effects through its ATG3-binding domains. Furthermore, KSHV impairs monocyte differentiation into dendritic cells by inhibiting autophagy, a process crucial for DC formation. KSHV reduces the expression of CAST (calpastatin), leading to decreased ATG5 levels in both THP-1 monocytic cells and primary monocytes [133]. These mechanisms represent how KSHV evades immune control by manipulating cellular autophagy.

Other mechanisms of evasion from the immune system by EBV include interference with the pathways for antigen presentation [134,135]. This defect, therefore, allows the virus to escape detection and elimination by the host’s immunity. Infection by EBV possibly decreases the number of MHC molecules on the surfaces of the infected cells [136,137]. MHC Class I presents endogenous antigens to CD8^+^ CTLs, while MHC Class II presents exogenous antigens to T helper cells or CD4^+^ [138]. Consequently, the downregulation of these MHC Classes I and II restricts the recognition and response of the immune system against the cells that have been taken over by EBV infection. EBV proteins have been known to interfere with the processing and presentation of viral antigens [139,140]. Studies have shown that EBNA-1 slows down the proteasomal degradation of cellular proteins and, hence, the presentation of endogenous antigens via MHC class I molecules, leading to the modulation of antigen presentation by viruses. The modulation of immune cell function by EBV acts directly on the function of immune cells. These are key processes for nasopharyngeal carcinoma and Burkitt’s lymphoma [120,134,135,141]. EBV has developed various mechanisms for modulating the crosstalk between immune cells to allow its evasion from immune surveillance and support its persistence [121,122]. EBV impacts a number of CTL-related signaling pathways. For example, EBV-expressed LMP-1 alters CTL signaling, which finally results in decreased CTL recognition and killing of the infected cells, an effect observed in both Hodgkin’s lymphoma and post-transplant lymphoproliferative disorder (PTLD). It leads to the maturation of regulatory T cells, which down-modulate immunological responses. EBV infection may be exploited for the induction of Treg in a bid to inhibit immune surveillance and to allow an environment that permits its persistence [120].

The LANA protein of KSHV impairs the presentation of viral antigens through MHC class I molecules and further impairs CD8+ T-cell recognition and cytolysis [142]. Such interference enables the virus to evade the adaptive immune response and, subsequently, to proliferate within the host. KSHV can evade immune surveillance through checkpoint pathways. These checkpoints refer to the regulators of the immune response that are crucial to maintain anti-autoreactivity. These mechanisms may be used by KSHV to modulate checkpoint pathways, especially the axis of PD-1/PD-L1, towards the inhibition of T-cell activation and the induction of immunological tolerance [143]. Indeed, it has been documented that KSHV efficiently inhibits T-cell-mediated cytotoxicity through the upregulation of PD-L1 in the infected cells, fostering long-term persistence [126,144,145]. KSHV infection induces functional impairments in diverse immune cells, including DCs and NK cells. The infection of DCs by KSHV inhibits cytokine secretion and antigen presentation, thereby inhibiting the activation of T lymphocytes [146]. In the same way, infection of NK cells by KSHV may impair their cytotoxic function and, hence, further reduce cytokine production and contribute to immune evasion and persistence [147].

EBV-infected cells produce a number of cytokines, some of which may have the ability to modulate the functions of immune cells. Thus, IL-10 produced by EBV-infected cells may downregulate both activation and functional activities from wide arrays of immune cells, including CTL and macrophages [121,122,148,149]. This mechanism plays a key role in EBV-associated gastric carcinoma. KSHV has developed multiple strategies to dampen innate cellular immune responses that are mediated through TLRs [150]. TLRs are important for recognizing viral components and, therefore, start an immune response. Indeed, KSHV expresses viral proteins, such as vIRF-1 and vIRF-2, that specifically target and inhibit TLR signaling pathways [151]. Thus, vIRF-1 inhibits TLR-3-induced activation by interfering with IRF-3 function, which is required to produce both Type I IFNs and inflammatory cytokines. KSHV inhibits the secretion of pro-inflammatory cytokines TNF-α, IL-1β, IL-6, and IFN-β mediated by TLR-4 and TLR-2 activation [150]. Furthermore, KSHV-encoded ORF63 has been demonstrated to reduce inflammasome activation through caspase-1 inhibition and the reduction of IL-1β and IL-18 levels in cells, which impairs the inflammasome activation of NLRP1 and NLRP3 and, thus, attenuates the inflammasome responses that these receptors typically drive [152,153]. The KSHV ORF52 protein has been found to block the initiation of RLR signaling and downstream signaling events through the MAVS-IRF-3 pathway to induce IFN-β expression [154]. RIG-I and MDA5 are the critical RLRs that recognize viral RNA and initiate antiviral immune responses. KSHV exploits the deubiquitinase activity of ORF64 to suppress RIG-I, thereby blocking the activation of IFN-β [155]. Due to these activities, the virus escapes all host immune systems and establishes a chronic infection completely. Similarly, KSHV also subverts cytoplasmic DNA sensing to escape immunity. For instance, KSHV-encoded LANA abrogates the action of one important cytosolic DNA sensor, cGAS [125,126,151,156]. Therefore, this interaction suppresses cGAMP generation via STING activation and further blocks type I IFN responses. Meanwhile, the KSHV ORF52 specifically impedes the production of cGAMP by the cGAS-STING pathway. The viral protein vIRF1 also antagonizes the interaction of TBK1 with STING, further inhibiting the cGAS-STING pathway and dampening IFN-I responses [157,158]. KSHV interferes with the host’s antigen presentation pathways to escape T-cell detection and evade adaptive immune responses [126,144]. KSHV encodes one of the largest numbers of proteins shown to have the potential to manipulate the presentation of viral antigens by MHC components. A thorough understanding of such mechanisms provides insights into the development of targeted therapies for virus-associated malignancies, which are especially focused on disrupting viral immune evasion strategies.

## 4. Cancers by EBV and KSHV

**Figure 5:**
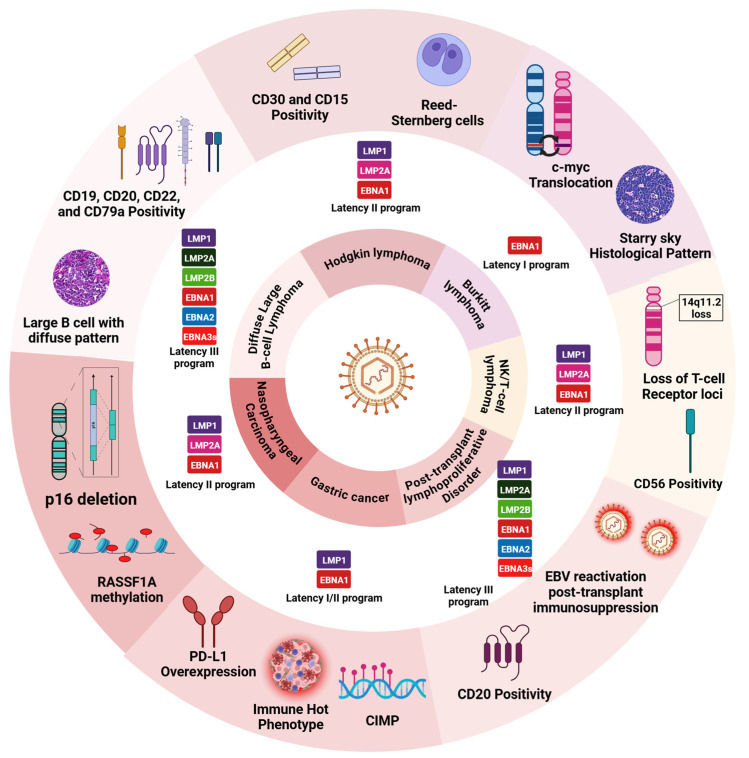
Epstein–Barr virus (EBV)-associated malignancies and latency. Hodgkin’s lymphoma is characterized by CD30/CD15 positivity and the presence of Reed–Sternberg cells. Burkitt’s lymphoma is distinguished by C-myc translocation and a starry sky histological pattern. Diffuse large B-cell lymphoma presents with large B cells along with CD19, CD20, and CD22 positivity. Nasopharyngeal carcinoma is marked by p16 deletion and RASSF1A methylation, while gastric cancer exhibits PD-L1 overexpression and an immune host phenotype. NK/T-cell lymphoma features CD56 positivity alongside the loss of T-cell receptor loci. Post-transplant lymphoproliferative disorder involves EBV reactivation under conditions of immunosuppression. Latency I, II, and III programs are associated with these malignancies as shown.

EBV is etiologically linked to several lymphoid and epithelial cancers, including Burkitt’s lymphoma, Hodgkin’s lymphoma, and nasopharyngeal carcinoma (Figure 5). KSHV, on the other hand, is predominantly associated with Kaposi’s sarcoma, primary effusion lymphoma, and multicentric Castleman disease [159]. Understanding the cancers caused by EBV and KSHV, as well as their oncogenic pathways, is crucial for developing prevention strategies. This section aims to provide a comprehensive overview of the malignancies associated with these viruses, highlighting their individual and combined roles in human cancer development.

### 4.1. Cancers Distinct and Common to EBV and KSHV

#### 4.1.1. Hodgkin’s Lymphoma

Hodgkin’s lymphoma, first described by Thomas Hodgkin in 1832, is a malignancy of the lymphatic system characterized by the presence of Reed–Sternberg cells [160,161]. It accounts for approximately 10% of all lymphomas and demonstrates a bimodal age distribution, with peaks in young adults and those over 55 years [162,163,164]. The disease is classified into two main types, namely classical Hodgkin’s lymphoma (cHL), which comprises about 95% of cases, and nodular lymphocyte-predominant Hodgkin’s lymphoma (NLPHL) [165,166]. The pathogenesis of Hodgkin’s lymphoma involves complex interactions between Reed–Sternberg cells and the surrounding microenvironment [167,168]. Reed–Sternberg cells secrete cytokines and chemokines that recruit and activate inflammatory cells, creating a supportive milieu for tumor growth [169]. This includes the attraction of T-helper 2 (Th2) and T-regulatory cells, which can suppress anti-tumor immune responses [170,171,172].

#### 4.1.2. Burkitt’s Lymphoma

Burkitt’s lymphoma is an aggressive B-cell lymphoma characterized by the rapid proliferation of malignant cells [173]. First described by Denis Burkitt in 1958 in African children, it has since been recognized as one of the fastest-growing human malignancies [174,175]. The lymphoma is classified into three clinical variants, namely endemic, sporadic, and immunodeficiency-associated [176]. The endemic form, highly prevalent in Equatorial Africa and Papua New Guinea, is strongly associated with EBV infection, with EBV present in nearly all cases [177,178]. Sporadic Burkitt’s lymphoma has a worldwide occurrence and is the most common form of BL outside of the malaria-infected zone [179,180]. The immunodeficiency-associated variant, often seen in HIV-positive individuals, shows significant EBV association [181]. All forms of Burkitt’s lymphoma are characterized by chromosomal translocations of t(8;14) (q24.1; q32) involving the c-MYC oncogene, and its variants, t(2;8) (p12; q24.1) and t(8;22) (q24.1; q11.2) [182]. These translocations juxtapose c-MYC with immunoglobulin heavy or light chain loci, leading to its constitutive overexpression and subsequent uncontrolled cell proliferation [183]. The role of EBV in Burkitt’s lymphogenesis is complex, exhibiting a unique type I latency program involving the expression of EBNA1, EBERs, and BART miRNAs [184,185,186]. EBV in Burkitt’s lymphoma provides a survival advantage to proliferating cells due to c-MYC translocation rather than directly driving proliferation throughout. This ‘hit-and-run’ mechanism allows EBV to contribute to lymphomagenesis while maintaining a minimal viral gene expression profile, evading immune detection [187]. The combination of c-MYC translocations and specific viral mechanisms results in the characteristic rapid proliferation of Burkitt’s lymphoma. Histologically, Burkitt’s lymphoma presents a “starry sky” pattern due to numerous mitotic figures and apoptotic cells [188]. The tumor typically involves extranodal sites, with the jaw and facial bones being common locations in the endemic form, while the abdomen, especially the ileocecal region, is more frequently affected in sporadic cases [189,190].

#### 4.1.3. Nasopharyngeal and Gastric Carcinoma

Nasopharyngeal carcinoma (NPC) is a unique epithelial malignancy arising from the nasopharynx, the upper part of the throat behind the nose [191]. It is characterized by its extremely unbalanced geographical distribution, particularly prevalent in Southeast Asia, Southern China, and North Africa [192,193]. The association between EBV and NPC is one of the strongest virus–cancer relationships known in endemic regions of NPC, where EBV contributes to 95% of NPC incidences and 100% of NPC-related mortalities with nearly [194]. In NPC, EBV exhibits a type II latency program, which is similar to Hodgkin’s lymphoma, but with some crucial differences in its oncogenic mechanisms [195]. EBV infection in NPC is believed to be an early, initiating event in carcinogenesis rather than a later step, as in some other EBV-associated cancers [196]. The nasopharyngeal epithelium provides a unique microenvironment that influences EBV–host interactions. The constant exposure to environmental factors (such as dietary nitrosamines) may contribute to genetic alterations that synergize with EBV infection [197]. NPC shows a distinct pattern of genetic alterations compared to other EBV-associated cancers, including the frequent inactivation of tumor suppressor genes like p16 and RASSF1A through promoter hypermethylation [198,199].

Gastric carcinoma is one of the leading causes of cancer-related deaths worldwide, with an exceptionally high incidence in East Asia [200]. While the majority of gastric cancer cases are associated with Helicobacter pylori infection, approximately 10% of cases globally are linked to Epstein–Barr virus (EBV) infection. This subset, known as EBV-associated gastric carcinoma (EBVaGC), represents a distinct molecular subtype of gastric cancer with unique characteristics [201]. EBVaGC is recognized by The Cancer Genome Atlas (TCGA) as one of four major genomic subtypes of gastric adenocarcinoma. This subtype is characterized by its specific clinicopathological features, molecular profile, and prognosis, distinguishing it from other forms of gastric cancer [202]. The prevalence of EBVaGC varies geographically, with higher rates observed in the Americas and lower rates in Eastern Europe. Interestingly, EBVaGC shows a male predominance and is more frequently found in the proximal stomach, particularly in the cardia and fundus [203]. In EBVaGC, the virus exhibits a latency I/II program, similar to nasopharyngeal carcinoma but distinct from other EBV-associated cancers. A hallmark of EBVaGC is extensive DNA hypermethylation, often referred to as the CpG island methylator phenotype (CIMP). This epigenetic alteration leads to the silencing of various tumor suppressor genes, including CDKN2A (p16) [204,205]. EBVaGC also exhibits distinct genetic alterations, like PIK3CA mutations, which occur in many cases. These mutations, along with the activation of the JAK2/PD-L1 signaling pathway, contribute to the unique molecular landscape of EBVaGC [206]. The immune microenvironment of EBVaGC is characterized by high levels of immune cell infiltration, particularly CD8+ T cells and macrophages [207,208]. This “immune-hot” phenotype, associated with elevated expression of PD-L1, indicates potential susceptibility to immune checkpoint inhibitors [209].

#### 4.1.4. Diffuse Large B-Cell and NK/T-Cell Lymphoma

Diffuse large B-cell lymphoma (DLBCL) is the most common type of non-Hodgkin’s lymphoma, accounting for approximately 30–40% of all cases worldwide [210]. It is characterized by the rapid proliferation of large neoplastic B cells and can arise de novo or as a transformation from a less aggressive lymphoma [190]. EBV-positive DLBCL was initially described in elderly patients, leading to the term “EBV-positive DLBCL of the elderly”. However, it is recently recognized that EBV-positive DLBCL can also occur in younger patients, prompting a revision in the World Health Organization classification to “EBV-positive DLBCL, not otherwise specified (NOS)” [211,212].

Extranodal NK/T-cell lymphoma, nasal type (ENKTL), is a rare but aggressive form of non-Hodgkin’s lymphoma characterized by its strong association with Epstein–Barr virus (EBV) infection [213]. This malignancy primarily affects the nasal cavity and upper aerodigestive tract, though extra-nasal sites can also be involved [214]. ENKTL demonstrates a distinct geographical distribution, being more prevalent in East Asia and parts of Central and South America and correlating with the epidemiology of EBV infection in these regions [215]. In ENKTL, the virus targets NK cells or cytotoxic T cells, leading to a unique pathogenesis.

#### 4.1.5. Post-Transplant Lymphoproliferative Disorder (PTLD)

Post-transplant lymphoproliferative disorder (PTLD) is a life-threatening complication that occurs in the context of immunosuppression following solid organ or hematopoietic stem cell transplantation. PTLD is strongly associated with Epstein–Barr virus (EBV) infection, with up to 70 of cases being EBV positive, particularly in early-onset PTLD occurring within the first year post-transplant [216,217]. The pathogenesis of PTLD is linked to EBV biology and the iatrogenic immunosuppression necessary for preventing graft rejection. In immunocompetent individuals, EBV-specific cytotoxic T lymphocytes (CTLs) effectively control the proliferation of EBV-infected B cells. However, in transplant recipients, immunosuppressive therapy impairs this T-cell-mediated immune surveillance, allowing for unchecked proliferation of EBV-infected B cells and leading to PTLD [218,219,220,221]. The clinical presentation of PTLD is highly variable, ranging from nonspecific symptoms like fever and weight loss to organ dysfunction due to tumor involvement [222]. The disease can be localized or disseminated, with common sites including lymph nodes, the gastrointestinal tract, and the central nervous system [223]. PTLD is classified into early lesions, polymorphic PTLD, monomorphic PTLD, and classical Hodgkin’s lymphoma-type PTLD [224].

#### 4.1.6. Kaposi’s Sarcoma

Kaposi’s sarcoma (KS) is a multifocal angio-proliferative disorder characterized by the abnormal growth of blood vessels, resulting in distinctive skin lesions and the potential involvement of internal organs. This malignancy is uniquely and universally associated with KSHV, also known as human herpesvirus 8 (HHV-8) [225]. The discovery of KSHV in 1994 revolutionized our understanding of KS pathogenesis and established it as one of the clearest examples of virus-induced human cancer [226]. KSHV prevalence varies widely geographically, with high rates in Sub-Saharan Africa and parts of the Mediterranean and lower rates in Northern Europe and Asia [44,227]. Kaposi’s sarcoma is classified into four epidemiological forms, namely classic KS, endemic KS, iatrogenic KS, and AIDS-associated KS. While all forms are caused by KSHV, the clinical presentation and course can vary significantly depending on the patient’s immune status and geographical location [228]. In KS lesions, KSHV primarily infects endothelial cells, transforming them into the characteristic spindle cells that are the hallmark of KS [229].

#### 4.1.7. Primary Effusion Lymphoma

Primary effusion lymphoma (PEL) is a rare and aggressive form of non-Hodgkin’s lymphoma characterized by its unique presentation as malignant effusions in body cavities without detectable tumor masses and is also universally associated with KSHV [230]. PEL typically occurs in immunocompromised individuals, particularly those with HIV/AIDS, although cases in immunocompetent individuals have been reported [231]. The disease most commonly affects serous cavities, including the pleural, pericardial, and peritoneal spaces [232]. In rare cases, solid variants of PEL can occur, presenting as extra-cavitary masses [233]. Interestingly, while KSHV is present in all cases of PEL, approximately 90% of cases are also co-infected with the Epstein–Barr virus (EBV). This dual infection suggests a complex interplay between these two oncogenic herpesviruses in the pathogenesis of PEL [234]. In PEL cells, KSHV displays a latent infection pattern, expressing a limited set of viral genes similar to those seen in Kaposi’s sarcoma [235]. LANA plays a crucial role in maintaining the viral episome and interfering with p53 and Rb tumor suppressor pathways [236]. vFLIP activates NF-κB signaling, promoting cell survival and proliferation, while vCyclin deregulates the cell cycle [237,238]. The virus induces a distinct gene expression profile in PEL cells, characterized by the activation of the NF-κB, JAK/STAT, and PI3K/AKT signaling pathways. EBV in PEL cells typically exhibits a restricted latency I program, expressing only EBNA1, EBERs, and BART miRNAs. While this expression pattern is more limited compared to KSHV, EBV still contributes significantly to PEL pathogenesis. The coinfection of KSHV and EBV in PEL creates a unique molecular environment. KSHV appears to be the primary oncogenic driver, but EBV enhances the transforming potential of KSHV-infected cells. The two viruses may cooperate in modulating cellular signaling pathways, particularly NF-κB and JAK/STAT, leading to enhanced cell proliferation and survival. EBV infection is reported to enhance the KSHV genome maintenance in PEL [239]. KSHV- and EBV-encoded miRNAs also play a significant role in PEL pathogenesis, modulating both viral and cellular gene expression to promote lymphoma cell survival and immune evasion [240].

### 4.2. EBV, KSHV and Childhood Cancers

Childhood cancer refers to cancers that occur in children, typically before the age of 20, and often involve rapidly growing tissues, such as in the blood, brain, or lymphatic system [241]. Childhood cancers in low- and middle-income regions show significant viral contributions, and in certain areas of the world, notably Sub-Saharan Africa and parts of Asia, these viruses are linked to a range of pediatric malignancies [242]. These cancers are often aggressive and challenging to treat due to limited healthcare infrastructure and delayed diagnosis. EBV is strongly implicated in several childhood cancers, most notably Burkitt’s lymphoma (BL) and Hodgkin’s lymphoma (HL), as well as EBV-positive T-cell and NK-cell lymphoproliferative diseases, with BL being the most prominent pediatric cancer associated with the EBV [243]. Though most EBV infections are asymptomatic or result in mild conditions like infectious mononucleosis, EBV can contribute to oncogenesis in immunocompromised individuals or regions with co-factors such as malaria [244,245,246]. The incidence of endemic Burkitt’s lymphoma (eBL) is as high as 3 to 6 per 100,000 children in areas such as Sub-Saharan Africa and Papua New Guinea [247]. On the other hand, up to 50% of childhood HL cases are EBV-positive in developing regions [248,249,250,251,252,253]. Clinically, children often present with fevers, night sweats, weight loss, and lymphadenopathy [254]. The survival outcomes for pediatric patients with EBV-positive HL are generally good, but long-term follow-up is critical to monitor for potential treatment-related complications, such as secondary malignancies or growth disturbances due to radiation therapy. But despite the good survival rates, the sequelae of therapy, such as altered somatic growth, infertility, and restrictive lung disease, seriously affect the quality of life of HL survivors [255]. In addition to BL and HL, EBV-associated T-cell and NK-cell lymphoproliferative disorders are rare but highly aggressive conditions in children. These diseases predominantly affect children and adolescents in certain regions, particularly in Asia and the Indigenous populations of Mexico, Central, and South America. In over 90% of cases, the disorders are linked to EBV, making it particularly significant in the context of childhood cancers in endemic regions [256,257,258]. The most common disorders in this group include chronic active EBV infection (CAEBV) of T- or NK-cell types, systemic EBV-positive T-cell lymphoma of childhood (STCLC), Hydroa vacciniforme (HV)-like lymphoproliferative disorder, and severe mosquito bite allergy (SMBA) [7]. Children with CAEBV suffer from persistent or intermittent symptoms that resemble infectious mononucleosis (IM), such as fever, fatigue, and lymphadenopathy, lasting for over three months. The disease progresses when the immune system fails to suppress the initial EBV infection, leading to high viral loads and multiorgan involvement, with the presence of EBV RNA or proteins in affected tissues [259,260]. Over time, CAEBV can evolve into more aggressive cancers, such as extranodal NK/T-cell lymphoma (ENKTL) or systemic EBV-positive T-cell lymphoma of childhood (STCLC), both of which are fatal if not treated early [261]. Hydroa vacciniforme (HV)-like lymphoproliferative disorder presents in children with papulovesicular lesions that worsen after sun exposure and may evolve into blisters and scarring. While mild cases resolve with photoprotection, severe forms can transform into aggressive lymphomas like ENKTL or STCLC [258,262]. Similarly, severe mosquito bite allergy (SMBA) displays itself as a hypersensitive reaction to mosquito bites, leading to local bullae and ulcers, with children showing high IgE levels, elevated NK-cells, and a significant EBV viral load [263,264]. Children with SMBA are at an increased risk of developing hemophagocytic syndrome and ANKL, and half of SMBA patients were reported to have died of hemophagocytic syndrome or leukemia/lymphomas [258]. Among these disorders, STCLC is the most aggressive. Children with STCLC rapidly develop fever, hepatosplenomegaly, cytopenias, and severe systemic inflammation [265]. The prognosis is poor, as most children succumb to the disease within days to weeks of diagnosis due to multiorgan failure [266]. In rare cases, early intervention with chemotherapy and stem cell transplantation has improved outcomes, but overall survival remains low [267,268].

Pediatric Kaposi sarcoma (KS), caused by KSHV, is one of the common childhood cancers prevalent in Eastern and Central Africa, particularly in areas with a high incidence of HIV [269]. Children with HIV infection are significantly more susceptible to KS, with a median incidence rate of 67.35 per 100,000 HIV-infected children in Sub-Saharan Africa, where over 90% of the world’s HIV-positive children reside [270,271,272]. KS in children often presents differently from adults, with primary lymphadenopathic KS being the most common form. This is characterized by bulging lymphadenopathy and sparse cutaneous lesions and is often accompanied by severe cytopenias, such as anemia and thrombocytopenia, particularly in cases involving visceral disease [273]. While ART has significantly reduced mother-to-child transmission of HIV, KS still occurs in both ART-treated and untreated children [274,275]. A major challenge is that pediatric KS, especially in its lymphadenopathic form, can present without typical hyperpigmented skin lesions, complicating diagnosis [276,277,278,279]. Children with severe lymphadenopathic KS frequently suffer from life-threatening cytopenias, with hemoglobin levels less than 8g/Dl in most cases to as low as 4 g/dL, and platelet counts under 10 × 10^9^/L in severe cytopenias, which often leads to delayed biopsies and treatment due to bleeding risks [279,280,281,282]. In addition to KS, KSHV is implicated in several other pediatric malignancies, though these are rarer. The most significant among them are multicentric Castleman disease (MCD), KSHV inflammatory cytokine syndrome (KICS), and primary effusion lymphoma (PEL). These conditions often occur in the setting of immunosuppression, particularly in children with HIV, and may present overlapping clinical features [144,283]. Multicentric Castleman disease (MCD) is a rare but serious lymphoproliferative disorder associated with KSHV, typically seen in immunocompromised children [284]. It is characterized by widespread lymphadenopathy, splenomegaly, and systemic inflammation, driven by high levels of cytokines such as IL-6 [285,286]. MCD shares overlapping clinical features with lymphadenopathic KS, such as severe cytopenias, making diagnosis difficult. However, unlike KS, MCD often presents with systemic inflammation, which helps differentiate between the two conditions. KSHV inflammatory cytokine syndrome (KICS), another KSHV-associated complication, presents with features similar to MCD, including hyperinflammatory syndrome with elevated IL-6 and IL-8 but without the histological presence of Castleman disease [286,287]. Viral IL-6, a homolog to human IL-6 is also elevated in the setting of KSHV lytic activation. Children with KICS typically exhibit fevers, hepatosplenomegaly, and elevated KSHV viral loads [273,288]. KICS is a serious condition in children that may lead to fatality as a consequence of multiorgan failure and is often associated with unresponsiveness to BV chemotherapy [288]. Pediatric cancers also include primary effusion lymphoma (PEL) associated with KSHV, typically presenting with malignant effusions in the pleural, peritoneal, or pericardial cavities without a solid tumor mass [49,289]. PEL is more commonly seen in HIV-positive adults and has been reported in pediatric patients, particularly those co-infected with HIV and KSHV [235].

### 4.3. EBV and KSHV in HIV Infection

The prevalence of EBV and KSHV infections is significantly higher in individuals with HIV compared to the general population, with these viruses contributing to a higher incidence of cancer and tumor development in HIV-positive patients [290,291]. HIV-induced immunosuppression creates an environment conducive to the reactivation of these viruses, which accelerates disease progression and elevates morbidity and mortality rates [292]. In individuals with HIV, there is an observed elevation in antibody titers against EBV antigens, an increased EBV DNA copy number in peripheral blood mononuclear cells, and heightened viral shedding in oropharyngeal secretions [293]. HIV infection is characterized by a progressive depletion of CD4+ T cells, which compromises the immunological surveillance necessary to control the herpesviruses’ latency [294]. Due to immunological dysregulations, T-cell responses become impaired, and EBV and KSHV are not effectively cleared, which prolongs their persistence and occasionally triggers their reactivation [142]. HIV infection induces pronounced B-cell activation, which, in the case of EBV co-infection, leads to the reactivation of latent EBV and subsequent EBV shedding [295,296].

Among the two viruses, Kaposi’s sarcoma (KS) was recognized as an AIDS-defining illness by the Centers for Disease Control and Prevention (CDC) in 1982 [228]. Diseases like non-Hodgkin’s lymphoma and invasive cervical cancer are often caused due to KS, which made it one of the three AIDS-defining cancers of the revised AIDS classification of 1993 [297]. KSHV, the causative agent of KS, manifests primarily in the context of severe immunodeficiency, such as that seen in HIV infection or immunosuppressive therapy. Despite the significant reduction in KS incidence over the past 25 years due to the introduction of effective antiretroviral therapy (ART), which restores immune function, people living with HIV (PLHIV) continue to have a risk of developing KS that is more than 30 times higher than in the general population, even in those with immune restoration [298]. 

The viral proteins from Kaposi’s sarcoma-associated herpesvirus, the human immunodeficiency virus, and the Epstein–Barr virus could be interacting with other viral proteins, as well as host cellular factors constituting a web of interactions that hold some important implications for viral replication, host cell function, and disease progression. At the heart of this elaborate machinery is HIV-1 tat, an extremely potent regulatory protein central to HIV replication. HIV-1 tat interacts with several other viral and cellular proteins that modulate a wide variety of cellular events. It cooperates with KSHV vGPCR in the activation of NF-κB [299,300]. In addition, HIV-1 tat synergizes with KSHV K1E-2, enhancing its actions [301]. Previous studies suggest that, in HeLa cells, HIV-1 tat is associated with cellular factors such as NF-AT, NF-AT1, and NF-AT2, which may modulate the gene expression that could influence viral persistence and replication [302]. KSHV proteins play several roles in this molecular play. KSHV vFlip, active in both 293T and Cos7 cells, probably pushes the infected cells to live through anti-apoptotic signaling [303]. KSHV K1E-2 activates HIV-1 tat and functions in BJAB, U937, and HEK293T cells, where it would seem to function in the induction of HIV-1 replication [301]. KSHV vGPCR upregulates NF-κB activity in primary KSHV-negative and KS-derived endothelial cells and may contribute to the inflammatory angiogenic phenotype seen in Kaposi’s sarcoma [303]. Similarly, the EBV proteins EBV BZLF-1, BRLF-1, and BamHI MLF-1 bind to NF-AT, NF-AT1, and NF-AT2 in HeLa cells, which presumably modulates host gene expression to favor viral replication and persistence [302,304,305]. EBV LMP-1 activates NF-κB and alters cellular signaling and survival pathways [306]. EBV EBNA-2, active in both B lymphocytes and HeLa epithelial cells, has an important role in the immortalization of B lymphocytes and could impact the function of epithelial cells [307].

**Figure 6:**
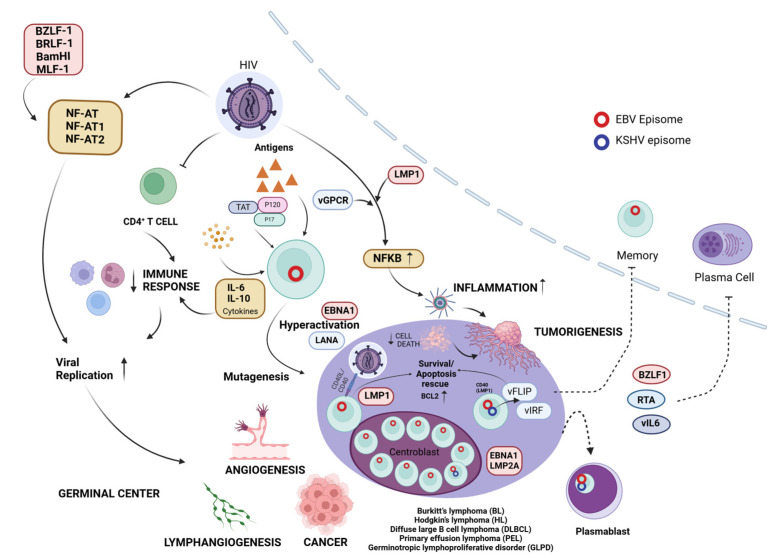
Molecular interplay between HIV, EBV, and KSHV in the context of immune dysregulation and oncogenesis. This figure illustrates the interplay between HIV, EBV, and KSHV in immune dysregulation and oncogenesis. It depicts how HIV infection compromises the immune system, particularly CD4+ T cells, creating an environment conducive to EBV- and KSHV-associated malignancies. EBV and KSHV, represented by their respective episomes, are shown to exploit the weakened immune system. Key viral proteins, such as LMP1 (EBV) and vGPCR (KSHV), activate NF-κB, a central mediator of inflammation and cell survival. Additionally, the figure shows how HIV with EBV viral proteins leads to the activation of NF-AT, NF-AT1, and NF-AT2, which are important transcription factors in T-cell activation and viral replication. These activations, along with other viral factors like EBNA1 and LANA, promote cell proliferation, inhibit apoptosis, and induce angiogenesis and lymphangiogenesis. The figure highlights the germinal center reaction, where EBV-infected B cells undergo hyperactivation and mutagenesis. This process, coupled with impaired immune surveillance, can lead to the development of various lymphomas, including Burkitt’s lymphoma, Hodgkin’s lymphoma, diffuse large B-cell lymphoma, and primary effusion lymphoma. The figure also illustrates the inhibition from infected cells to memory B cells and plasma cells, emphasizing the role of these viruses in manipulating B-cell differentiation and survival. Overall, this figure highlights the interactions between HIV, EBV, and KSHV, demonstrating how these viruses cooperatively subvert host immune responses, induce chronic inflammation, and drive lymphomagenesis.

This interaction of the viral protein with the cytosolic factor triggers several significant consequences, such as HIV-1 LTR activation, which is controlled by many regulators, including HIV-1 tat, IFN-γ, and KSHV K1E-2, and probably enhances HIV replication [308]. Such interactions may be correlated to increased transcriptional activity for HIV-1, as well as with the development of KS-like lesions in nude mice [299]. Viral–host interactions, overall, powerfully alter cellular processes. Apoptosis and the cell cycle are modified, and HIV-1 tat will play a central role in these cellular events [309]. Many of the viral proteins activate NF-κB to enhance survival and inflammation within the cell [307]. IL-6 and Jak/STAT3 signaling can be induced, which may also contribute to the inflammatory environment seen in viral infections [299,310]. These networks of protein–protein interaction depositions thereby reveal the interaction between viruses such as KSHV, HIV-1, and EBV, which also further outlines their effects on host cellular processes. The core position of HIV-1 tat in orchestrating so many such interactions raises the possibility that it may be an important factor in promoting both viral replication and pathogenesis [311,312]. Overall, the cumulative effects of such interactions seem to favor viral persistence, enhanced replication, and the conditions that are favorable for promoting the cellular environments that lead to viral-associated malignancies [310,313] (Figure 6).

It plays a very important role in the overall development of targeted therapies and interventions in the fight against these viral infections and their associated diseases. The coexistence of HIV, EBV, and KSHV in the same host can lead to synergistic interactions that exacerbate disease progression. HIV-induced immunosuppression not only allows for the reactivation of EBV and KSHV but also enhances their oncogenic potential. These interactions can influence viral reactivation, persistence, and the development of malignancies. The complex interplay between these viruses may also contribute to the atypical clinical presentations and diagnostic challenges observed in HIV-positive patients.

### 4.4. EBV, KSHV Co-Infection

Approximately 90% of PELs exhibit the presence of EBV alongside KSHV [237,314,315]. In PEL cell lines, co-infection with both viruses is common, with their genomes being maintained, replicated independently, and segregated into daughter cells [237,239,316]. In vitro studies have shown that, while KSHV can infect peripheral B cells independently, it is unable to transform them or sustain long-term infection on its own [317,318,319]. Evidence supporting the role of EBV in enhancing KSHV persistence comes from in vivo co-infection studies using humanized mice models, where dual infection significantly increases the likelihood of KSHV persistence [147,320]. EBV co-infection promotes B-cell stimulation and facilitates prolonged KSHV infection through transformation, which is dependent upon the expression of at least one EBV transforming gene [239,317]. The Epstein–Barr virus utilizes a suite of viral proteins to facilitate B-cell transformation and create conditions favorable for persistent infection. EBNA2, in conjunction with other viral proteins like EBNA-LP, EBNA3A, EBNA3C, and LMP1, plays a crucial role in this process [321]. EBNA2 primarily functions by stimulating the proliferation of infected cells. It achieves this by upregulating the expression of genes involved in cell cycle progression, such as cyclin-D2, cyclin-E, and c-myc [322]. EBNA-LP further enhances the role of EBNA2. While EBNA2 and EBNA-LP focus on cell proliferation, EBNA3A and EBNA3C contribute to the transformation process by inducing DNA damage. LMP1, another key player, serves a dual purpose by promoting both cellular transformation and proliferation [323]. Beyond its role in transformation and proliferation, LMP1 also enhances cell survival. It accomplishes this by modulating the NF-κB signaling pathway. Additionally, in the context of PEL, LMP1 exerts transcriptional control, leading to the intermittent expression of itself and various non-coding RNAs [324]. Collectively, these viral proteins work in concert to establish and maintain a cellular environment that supports persistent KSHV infection. This altered cellular state ultimately contributes to the emergence of PEL. Although the precise mechanisms remain unclear, co-infected cells appear to harbor a higher number of KSHV genomes per cell, suggesting a stabilizing effect of EBV on KSHV genome maintenance [320,325]. During co-infection, the expression of both viral genomes is downregulated to a latent state, with the EBV showing a reduced expression of its latent genes. KSHV and EBV employ distinct, yet complementary, immune evasion strategies to evade host immune surveillance. This synergy contributes to chronic inflammation, tissue damage, and an immunological environment conducive to tumorigenesis [326]. Notably, KSHV/EBV co-infection leads to robust T-cell activation, particularly of CD8+ T cells, indicating that both EBV- and KSHV-specific T cells are primed and expanded during infection [327]. Further investigation into EBV/KSHV co-infection, particularly in Kaposi’s sarcoma (KS), is required. Studies should include detailed clinical data, such as patient demographics, disease progression, prior medical history, and outcomes, to provide a comprehensive understanding of the clinical implications of this co-infection.

## 5. Antivirals and Anti-Tumor Therapeutics Against EBV and KSHV

### 5.1. Current Antiviral Therapies

The regulation of Epstein–Barr virus (EBV) and Kaposi’s sarcoma-associated herpesvirus (KSHV) infection is complex, in part due to the limitations of existing antiviral therapies. These viruses are persistent and associated with several severe diseases, making the development of effective treatment strategies essential. Acyclovir, Famciclovir, Ganciclovir, Cidofovir, and Maribavir are among the antiviral drugs commonly used, each with distinct mechanisms of action, efficacy profiles, and side effects.

Acyclovir, one of the earliest antiviral drugs developed specifically for herpesviruses, was approved by the FDA in 1982. It is a guanosine analog that targets viral replication by interacting with viral thymidine kinase, leading to chain termination during DNA synthesis [328,329]. Since the introduction of Acyclovir, substantial progress has been made to relieve patients suffering from viral infections [330]. Acyclovir is particularly effective against the herpes simplex virus (HSV) and the varicella-zoster virus (VZV). Although Acyclovir has shown many positive results over the years, it also has many side effects, primarily nausea, diarrhea, and headache, with rare cases having nephrotoxicity and neurotoxicity, which can lead to renal conditions due to uric acid precipitation. Valacyclovir is a prodrug form of acyclovir that was approved by the FDA in 1995. Valacyclovir is a significant variant of Acyclovir with improved bioavailability. Once ingested, Valacyclovir can rapidly be converted into acyclovir in the body, which in turn, interrupts the viral DNA synthesis process [331,332,333,334]. Long-term administration of Valacyclovir has been shown to significantly reduce the viral load in patients infected with the EBV [335]. Ganciclovir, originally developed for cytomegalovirus (CMV) infections, has proven to be effective against EBV, particularly in cases of PTLD [336,337]. Ganciclovir is also used prophylactically to reduce the risk of EBV-induced PTLD in solid organ transplant recipients, decreasing incidence by approximately 50% [338,339]. However, Ganciclovir has notable side effects, including neutropenia, anemia, thrombocytopenia, and possible nephrotoxicity [340]. Combination therapy with Ganciclovir and immune globulin has been explored to enhance its prophylactic efficacy against EBV-related PTLD [337].

Foscarnet, also known as phosphonoformate, was approved in 1989. It mimics pyrophosphate, a natural inhibitor of viral DNA polymerases, and prevents the elongation of viral DNA by directly inhibiting these polymerases [341]. While primarily used for herpesviruses, Foscarnet has shown potential in controlling EBV infection in transplant recipients with PTLD by enhancing cellular immunity [342]. Cidofovir, a nucleotide analog phosphorylated by cellular kinases, inhibits viral DNA polymerase by competing with native nucleotides, thereby integrating into viral DNA [343,344]. Cidofovir has demonstrated efficacy against EBV, reducing the presence of viral oncoproteins and increasing sensitivity to radiation in EBV-related malignancies [345,346]. It has been employed in treating recurrent EBV-associated nasopharyngeal cancer, with promising results in enhancing therapeutic outcomes. Imatinib, primarily used for chronic myeloid leukemia (CML) and gastrointestinal stromal tumors (GISTs), inhibits BCR-ABL tyrosine kinase, which is crucial for CML proliferation [347]. Imatinib is administered orally and is well-absorbed from the gastrointestinal tract. It is extensively metabolized in the liver, primarily by the cytochrome P450 enzyme CYP3A4. The most prevalent ocular side effects related to imatinib mesylate are Maculopapular eruptions, erythematous eruptions, edema, and periorbital edema [348,349]. Recent studies suggest that Imatinib shows promise beyond its initial indications, demonstrating potential in the treatment of the Epstein–Barr virus (EBV) and Kaposi’s sarcoma-associated herpesvirus (KSHV)-related malignancies due to its ability to target specific kinases that are implicated in these viral infections. Notably, Imatinib has shown efficacy in controlling chronic active EBV infections, with clinical outcomes improved by approximately 33% [350]. Maribavir, the newest antiviral agent approved by the FDA in 2021, is primarily used for CMV infections in transplant recipients [351]. Unlike the conventional antivirals that target viral DNA polymerase, Maribavir inhibits the viral protein kinase UL97, a crucial enzyme for CMV DNA replication. Maribavir is also being investigated for its potential in treating EBV-related disorders, especially in cases where resistance to existing antivirals is a concern [352,353]. Studies have shown promising results, suggesting that it could offer a new therapy option, especially to people resistant to existing medications [354].

Currently, no licensed antiviral drugs specifically target KSHV. The treatment of KSHV-associated diseases, such as Kaposi’s sarcoma, typically involves chemotherapy regimens, including liposomal doxorubicin or paclitaxel, to reduce tumor size and control disease progression. Targeted therapies, such as angiogenesis inhibitors, are also being investigated to impair the blood supply to KSHV-associated tumors [355]. Antiretroviral therapy (ART) plays a critical role in managing HIV, which is strongly associated with KSHV-related morbidity. By controlling HIV replication and restoring immune function, ART indirectly reduces the incidence and severity of KSHV-associated diseases [356]. Immunotherapies, such as cytokine-targeted therapeutics, are being explored to modulate immune responses against KSHV-associated malignancies [357,358]. The absence of direct antiviral treatments for KSHV underscores the need for further research. A phase I trial involving pomalidomide combined with liposomal doxorubicin has shown potential for treating Kaposi’s sarcoma, with or without other KSHV-associated diseases [359]. These ongoing studies highlight the urgent need for more effective and targeted therapies for KSHV and related conditions.

The antiviral drugs primarily target the lytic replication phase of EBV and KSHV infections. Lytic replication is an attractive target because it is associated with active viral replication, which these antivirals can interrupt [360]. Drugs like Acyclovir, Ganciclovir, and Foscarnet inhibit viral DNA synthesis or polymerases, processes critical to lytic replication. However, targeting viral latency remains a significant challenge. Latency allows the virus to persist in host cells without active replication, evading the immune system and existing antiviral treatments. Understanding the mechanisms of latency establishment and maintenance is crucial, as even low infectious doses can result in significant levels of latency, complicating therapeutic interventions [361]. Latent viral genes, such as EBNA (Epstein–Barr nuclear antigens) in EBV and LANA (latency-associated nuclear antigen) in KSHV, contribute to oncogenesis and are not affected by drugs targeting lytic replication. This underscores the need for therapies targeting the latency phase to effectively prevent associated malignancies and viral reactivation. Future research focusing on immunotherapies or live-attenuated vaccines to target latent virus reservoirs shows promise. However, translating these strategies into effective treatments remains a significant challenge.

### 5.2. Anti-Tumor Strategies for EBV and KSHV-Associated Cancers

#### 5.2.1. Monoclonal Antibodies

The anti-CD20 monoclonal antibody Rituximab has already been used with unquestionable success to treat EBV-associated lymphomas, which are predominantly differentiated by CNS involvement. Rituximab binds to CD20, marking the B cells for destruction via mechanisms like antibody-dependent cellular cytotoxicity (ADCC), complement-mediated cytotoxicity (CMC), and apoptosis. By selectively targeting these B cells, Rituximab depletes cancerous cells while sparing normal tissues. Mortality from PTLD has been reduced drastically in the allogeneic stem cell transplantation recipients who receive pre-emptive Rituximab. It has been shown that deaths before the introduction of pre-emptive use of Rituximab were 40% of all deaths caused by PTLDs compared to the post-pre-emptive use, at just an 11% mortality rate [362]. Brentuximab Vedotin is yet another antibody–drug conjugate targeted to CD30 and has been utilized in combination with Rituximab in the treatment of EBV-positive and CD30-positive lymphomas. Brentuximab binds to CD30 and is internalized by the cancer cell, and the attached drug (vedotin) is released, disrupting the microtubule network and leading to apoptosis. This selective action minimizes damage to normal tissues. Combination in phase I research, in conjunction with Rituximab, had an overall response rate of 88%, while 50% of patients attained full remission. In a phase II study of relapsed or refractory EBV-positive and CD30-positive lymphomas, the total response rate was established at 83%, whereas 61% of patients showed complete recovery [363,364]. Because the targeted therapies have a narrow spectrum of activity, these treatments enable the killing of almost all tumor cells with minimal damage to normal tissues. This combination allows for the targeted killing of tumor cells while sparing normal tissues, thanks to the focused action on CD30 and CD20.

Rituximab-targeting CD20+ B-cells, in combination with liposomal doxorubicin, produced excellent results among the HIV positive with KSHV-related MCD. In clinical trials, Rituximab, in combination with liposomal doxorubicin, achieved an overall response rate of 64% among those patients while markedly prolonging the time to progression of the disease and, therefore, enhancing survival for the cohort [365,366]. A phase II trial of Bevacizumab, an anti-VEGF monoclonal antibody, reported a partial response in 31% of those patients, and 44% were documented as having a stable disease when added to ART [367]. Bevacizumab blocks VEGF, a critical factor in angiogenesis, which is vital for KSHV-driven cancers like Kaposi’s sarcoma. By inhibiting blood vessel formation, it starves the tumor, halting its growth and spread. Another pilot study has been shown to have as significant as an overall response rate of 45% in advanced KS patients and that combining Bevacizumab with liposomal doxorubicin further increases anti-tumor efficacy [368].

#### 5.2.2. Immune Checkpoint Inhibitors

Immune checkpoint inhibitors are emerging considerations for EBV-associated lymphoproliferative disorders and lymphomas. For relapsed or refractory EBV-associated hemophagocytic lymphohistiocytosis and other forms of EBV-positive lymphomas, the PD-1 inhibitor Nivolumab is effective. Nivolumab blocks the PD-1/PD-L1 interaction, restoring the ability of T cells to recognize and attack cancer cells by preventing immune evasion. The overall response rate was observed at 61% in phase 2 with Nivolumab while controlling the disease in 89% of patients. Nivolumab blocks the interaction of PD-1 with its ligands and, thus, allows the immune system to recognize cancer cells again by restoring this function [369,370]. Similarly, the PD-1 inhibitor Pembrolizumab has been used in EBV-positive and EBV-negative lymphomas. Pembrolizumab works by the same mechanism, reactivating immune surveillance of tumors. A comparison of the efficacy of Pembrolizumab indicated a 60% overall response rate in the case of EBV-positive patients, while it was only a 40% overall response rate for EBV-negative cases, which again highlights the increased sensitivity of EBV-positive lymphomas toward immune checkpoint inhibition [371]. It has also been used and proven to be effective as a first-line treatment for newly diagnosed EBV-negative extranodal natural killer/T-cell lymphoma, showing suitability once again [372]. Rapamycin is a common mTOR (mechanistic target of rapamycin) inhibitor in cancer because of its ability to hamper the development and division process of cells. Rapamycin specifically inhibits the mTOR pathway, which is hyperactivated in viral-induced cancers like EBV. By inhibiting mTOR, Rapamycin slows cancer cell growth, reduces angiogenesis, and dampens immune evasion, resulting in smaller tumors and slower progression. Studies show Rapamycin to 50% inhibit mTOR signaling in EBV-associated nasopharyngeal cancer, resulting in a 45% decrease in tumor size [373].

Immunomodulatory agents like Pomalidomide and Lenalidomide have emerged as promising therapies for restoring immune function in KSHV-infected cells. These drugs boost immune surveillance by restoring the expression of immune surface markers on KSHV-infected cells, making them more susceptible to attack by T cells and NK cells. Additionally, they stimulate the production of cytokines, enhancing the immune system’s ability to control the tumor. In a clinical trial involving Pomalidomide for Kaposi’s sarcoma, HIV-positive and HIV-negative patients exhibited a 67% overall response rate, with 12% complete response and 55% partial response. Importantly, long-term follow-up demonstrated that 72% of responders remained progression-free after 12 months of treatment [374,375]. Moreover, it has been shown that both Lenalidomide and Pomalidomide can restore the expression of immune surface markers on infected cells with KSHV, thereby enhancing immune surveillance and targeting [374]. The mTOR inhibitor Everolimus proved to be extremely promising, both in terms of KS and primary effusion lymphoma (PEL). By inhibiting mTOR, Everolimus reduces the growth and survival signals of tumor cells, leading to reduced cell proliferation and angiogenesis. This results in tumor shrinkage and better immune control. There are case reports of a renal transplant patient who was given Everolimus. The systemic and cutaneous KS completely resolved within months of initiation of treatment [17]. A multi-targeted therapy with Everolimus also displayed an overall response rate of 50% in patients with PEL, implying that it is effective against both oncogenesis and angiogenesis caused by KSHV [376,377]. The inhibition of mTOR reduces neoangiogenesis and immunosuppression and, thus, would offer a holistic treatment method for KSHV-related cancers [378,379].

In addition to the proteasome inhibitors, Bortezomib is among the therapeutic drugs used in PEL, where KSHV drives malignancies. Bortezomib enhanced the reactivation of both KSHV and the EBV lytic cycle through the JNK signaling pathway that sensitized the tumor cells to therapeutic chemotherapy. Bortezomib inhibits the proteasome, leading to the accumulation of misfolded proteins, inducing stress, and triggering apoptosis in tumor cells. It also reactivates the KSHV and EBV lytic cycle, sensitizing the tumor cells to chemotherapy. In in vitro studies, the combination of Bortezomib and Metformin enhanced the apoptosis of PEL cells by up to 50% by improved cytotoxicity and enhanced the unfolded protein response of Metformin, which causes inhibition in the lytic cycle of KSHV [380,381]. The combination of monoclonal antibodies with immune checkpoint inhibitors has proven to be better for tumor treatment. Currently, the combination of Rituximab with immune-checkpoint inhibitors, such as Pembrolizumab or Nivolumab, is under research as an enhancement of immune reaction against EBV-positive lymphomas. This approach exploits the mechanism of targeted action of Rituximab against CD20+ B cells, along with bringing back the functionality of the immune system through the action of checkpoint inhibitors [370,371]. In diseases like stage IV NK/T-cell lymphoma with chronic active EBV, it has been treated as a combination of therapies in the form of Pembrolizumab and TCRαβ-depleted haploidentical hematopoietic stem cell transplantation. So, in many such cases, complete remission has been achieved and has underlined the feasibility and effectiveness of such a mode of treatment that targets both viral and tumor elements of the disease [372,382].

### 5.3. Vaccine Development Against EBV and KSHV

**Figure 7:**
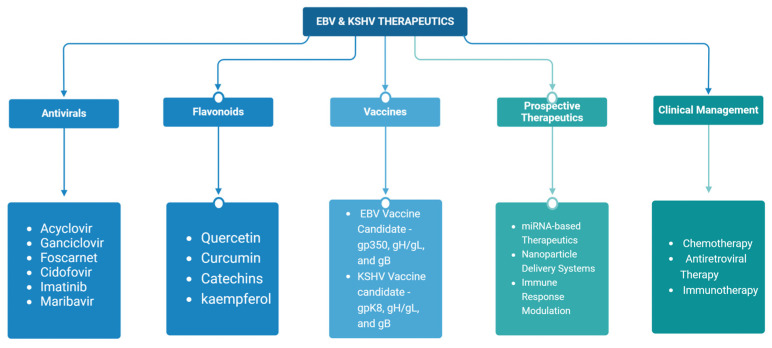
Antiviral mechanisms/therapeutic strategies for EBV and KSHV. This flowchart presents an overview of current and prospective therapeutic approaches for EBV- and KSHV-associated diseases. It outlines five main categories of interventions: antivirals, flavonoids, vaccines, prospective therapeutics, and clinical management strategies. The figure details specific examples within each category, such as acyclovir and ganciclovir as antivirals, quercetin and curcumin as flavonoids, and vaccine candidates targeting viral glycoproteins. It also highlights emerging therapies like miRNA-based therapeutics and nanoparticle delivery systems. The figure provides a comprehensive summary of the multifaceted approach to treating EBV and KSHV-related conditions, encompassing both established and innovative therapeutic strategies.

EBV has six main membrane glycoproteins, namely gp42, gp350, BMRF2, gH, gL, and gB. Of these, gp350 serves as the principal attachment protein (anti-receptor), while gp42, gH/gL, and gB function as fusion proteins that mediate viral entry into host cells [383]. In EBV vaccine development, gp350 has been prioritized due to its abundance on the viral envelope. However, studies have shown that, despite the presence of gp350-neutralizing antibodies, EBV infection can still occur, indicating that viral entry is not solely dependent upon gp350. When gp350 was combined with the fusion proteins gH/gL and gB, a synergistic effect was observed, resulting in significantly enhanced protection against EBV infection [384,385]. Various methods have been explored for delivering these glycoproteins, including mRNA vaccines and virus-like particles (VLPs). For targeting EBV during its latent phase, latent proteins, such as EBNA1, LMP1, and LMP2, are key candidates. These proteins can elicit specific CD4+ and CD8+ cytotoxic responses with anti-tumor potential. Both chimeric antigen receptor (CAR) T cells and cytotoxic T lymphocytes (CTLs) have been studied for EBV-associated cancers. In vitro-expanded EBV-specific CTLs have shown efficacy in preventing and treating post-transplant lymphoproliferative disorder (PTLD), while CAR T cells engineered to target LMP1 and LMP2 have demonstrated reduced tumor growth in both in vitro and animal studies [386,387,388]. Similarly, KSHV has four key glycoproteins, namely gpK8.1, gB, and gH/gL. In KSHV vaccine development, gpK8.1 is favored due to its abundance on the viral envelope. As with EBV, combining gpK8.1 with the fusion proteins gH/gL and gB resulted in enhanced efficacy against viral infection [389]. Figure 7 offers a thorough overview of the multifaceted treatment of KSHV- and EBV-related cancers, including both current and prospective therapeutic strategies (Figure 7).

Despite this progress, significant challenges remain in the development of vaccines against gamma herpesviruses like EBV and KSHV, primarily due to their oncogenic nature and complex life cycles. One major obstacle is the ability of these viruses to establish lifelong latency. Targeting latency-associated proteins for vaccine development raises concerns about disrupting essential cellular functions and potentially triggering adverse effects [390]. Another challenge lies in the complexity of viral entry mechanisms. These viruses utilize multiple entry pathways that are mediated by a combination of glycoproteins, such as gp350, gp42, gH/gL, and gB for EBV and gpK8.1, gH/gL, and gB for KSHV. Vaccines that target a single glycoprotein may not provide comprehensive protection.

Furthermore, these viruses employ sophisticated immune evasion strategies, including the downregulation of MHC molecules and the production of viral homologs of cytokines, which dampen immune responses and limit vaccine efficacy [391]. The need to simultaneously target both the lytic and latent phases of infection adds to the complexity, as the antigenic profiles and immune mechanisms required to control these phases differ significantly [392]. Moreover, gammaherpesviruses have sophisticated mechanisms to evade the immune system, complicating efforts to elicit robust and lasting immunity. Challenges include the poor immunogenicity of viral antigens and the difficulty of inducing protective antibody and T-cell responses without risking oncogenesis. The lack of suitable animal models that mimic human EBV and KSHV infections poses additional challenges for evaluating vaccine candidates. Moreover, the broad spectrum of diseases caused by these viruses, ranging from benign infections to aggressive cancers, complicates vaccine design, as a single formulation must address diverse clinical outcomes [76]. Finally, safety concerns arise when targeting latent proteins like EBNA1 or LANA, as this approach carries risks of autoimmunity or unintended oncogenic effects, further complicating the path to a viable vaccine [393]. Live attenuated or viral vector-based vaccines may pose risks of latent infections or reactivation in immunocompromised individuals.

Novel approaches, such as replication-deficient or inactivated virus vaccines, are under investigation and show promise in preclinical models by reducing latency and reactivation risks. However, the lack of FDA-approved vaccines for gammaherpesviruses underscores the need for innovative strategies and comprehensive clinical trials. These challenges highlight the need for innovative strategies that combine approaches to enhance vaccine efficacy while addressing the unique complexities of gamma herpesviruses.

## 6. Conclusions

Taken together, the complexity of the cancer-driving mechanisms of the EBV and KSHV, involving viral genetics, epigenetic alterations, and immune evasion strategies, has made these viruses a formidable challenge in oncology and virology. One major gap lies in the development of effective antiviral and anticancer therapies. The current treatments primarily target virus-associated malignancies post-oncogenesis, with limited success in eradicating latent viral infections or preventing viral reactivation. Therefore, the urgent need for strategies that can effectively target latent infections, prevent viral reactivation, and simultaneously avoid damage to normal host cells is the need of the hour. Furthermore, the lack of a comprehensive understanding of viral entry, tropism, and fusion hinders the development of more precise antiviral therapies and is an area ripe for further research. In light of the current therapeutics, a more integrated approach, combining antiviral and anticancer strategies, holds the potential to significantly reduce the burden of gamma herpesvirus-associated cancers.

## Figures and Tables

**Table 1:** Role of viral microRNAs (miRNAs) in cancer. This table summarizes the key viral microRNAs (miRNAs) encoded by Epstein–Barr virus (EBV) and Kaposi’s sarcoma-associated herpesvirus (KSHV), along with their molecular targets and functional roles in cancer development. These miRNAs play critical roles in immune evasion, apoptosis inhibition, angiogenesis, epithelial–mesenchymal transition (EMT), cell cycle regulation, and metabolic reprogramming, contributing to viral persistence and oncogenesis.

Virus	miRNA	Target	Function/Role
EBV	miR-BART5	PUMA	Promotes cell survival by inhibiting apoptosis
	miR-BART3-3p	BIM	Reduces production of pro-apoptotic proteins
	miR-BART5-5p	BIM, TP53	Promotes immune evasion and cell survival
	miR-BART2-5p	BIM	Assists in immune evasion
	miR-BART17-5p	TAP2	Prevents antigen processing and presentation
	miR-BHRF1-2-5p	IL-1R1	Inhibits immune system alertness to viral infections
	miR-BART6-3p	RIG-I, LOC553103	Inhibits innate immune response and suppresses EMT
	miR-BART8	STAT1	Reduces cellular immunity against tumor cells
	miR-BART20-5p	IFN-γ	Blocks immune signaling
	miR-BART15-3p	NLRP3	Suppresses IL-1β, IL-18 production, reducing inflammation
	miR-BART15-5p	BRUCE, TAX1BP1	Induces apoptosis in tumor and immune cells
	miR-BART14	lncRNA AFG3L1P	Promotes cell survival and mitochondrial dysfunction
	miR-BART22	MOSPD2 mRNA	Promotes EMT and metastasis via Wnt/β-catenin signaling
	miR-BART7-3p	PTEN, SMAD7	Promotes tumor growth, invasion, and CSC-like features
KSHV	miR-K12-11	MIR17HG, MIR155HG, MALAT1, AFAP1-AS1	Mimics miR-155 to exploit cellular regulatory networks
	miR-K12-10	TWEAKR	Promotes cell survival by inhibiting apoptosis
	miR-K1, K3-3p, K6-3p	Thrombospondin 1	Promotes angiogenesis by targeting anti-angiogenic factors
	miR-K6-5p	Not explicitly mentioned	Enhances endothelial cell tubulogenesis
	miR-K1, K3, K4-3p	Caspase 3	Inhibits apoptosis to maintain infected cell populations
	miR-K1	p21	Regulates cell cycle by inhibiting key cell cycle inhibitors
	miR-K10a, K10b	TGF-β signaling	Promotes cell survival by modulating differentiation and cell survival pathways
	miR-K6, K11	Differentiation factors of endothelial cells	Contributes to vascular tumor formation in KSHV-associated malignancies

## Data Availability

In this review article no new data were created.
